# Design, Synthesis, Chemical and Biochemical Insights Into Novel Hybrid Spirooxindole-Based p53-MDM2 Inhibitors With Potential Bcl2 Signaling Attenuation

**DOI:** 10.3389/fchem.2021.735236

**Published:** 2021-12-14

**Authors:** Yasmine M. Abdel Aziz, Gehad Lotfy, Mohamed M. Said, El Sayed H. El Ashry, El Sayed H. El Tamany, Saied M. Soliman, Marwa M. Abu-Serie, Mohamed Teleb, Sammer Yousuf, Alexander Dömling, Luis R. Domingo, Assem Barakat

**Affiliations:** ^1^ Pharmaceutical Organic Chemistry Department, Faculty of Pharmacy, Suez Canal University, Ismailia, Egypt; ^2^ Department of Chemistry, Faculty of Science, Alexandria University, Alexandria, Egypt; ^3^ Department of Chemistry, Faculty of Science, Suez Canal University, Ismailia, Egypt; ^4^ Medical Biotechnology Department, Genetic Engineering and Biotechnology Research Institute, City of Scientific Research and Technological Applications (SRTA-City), Alexandria, Egypt; ^5^ Department of Pharmaceutical Chemistry, Faculty of Pharmacy, Alexandria University, Alexandria, Egypt; ^6^ H.E.J. Research Institute of Chemistry, International Center for Chemical and Biological Sciences, University of Karachi, Karachi, Pakistan; ^7^ Department of Drug Design, Groningen Research Institute of Pharmacy, University of Groningen, Groningen, Netherlands; ^8^ Department of Organic Chemistry, University of Valencia, Valencia, Spain; ^9^ Department of Chemistry, College of Science, King Saud University, Riyadh, Saudi Arabia

**Keywords:** spirooxindole, protein–protein interaction (PPI), p53, human homolog of mouse double minute 2 (MDM2), Bcl 2

## Abstract

The tumor resistance to p53 activators posed a clinical challenge. Combination studies disclosed that concomitant administration of Bcl2 inhibitors can sensitize the tumor cells and induce apoptosis. In this study, we utilized a rapid synthetic route to synthesize two novel hybrid spirooxindole-based p53-MDM2 inhibitors endowed with Bcl2 signaling attenuation. The adducts mimic the thematic features of the chemically stable potent spiro [3*H*-indole-3,2′-pyrrolidin]-2(1*H*)-ones p53-MDM2 inhibitors, while installing a pyrrole ring *via* a carbonyl spacer inspired by the natural marine or synthetic products that efficiently inhibit Bcl2 family functions. A chemical insight into the two synthesized spirooxindoles including single crystal x-ray diffraction analysis unambiguously confirmed their structures. The synthesized spirooxindoles **2a** and **2b** were preliminarily tested for cytotoxic activities against normal cells, MDA-MB 231, HepG-2, and Caco-2 *via* MTT assay. **2b** was superior to 5-fluorouracil. Mechanistically, **2b** induced apoptosis-dependent anticancer effect (43%) higher than that of 5-fluorouracil (34.95%) in three studied cancer cell lines, activated p53 (47%), downregulated the Bcl2 gene (1.25-fold), and upregulated p21 (2-fold) in the treated cancer cells. Docking simulations declared the possible binding modes of the synthesized compounds within MDM2.

## Introduction

The World Health Organization declared in its 2020 report the burden posed by cancer worldwide. Two years ago, the estimated annual cancer cases were 18.1 million including 9.6 million deaths. These figures are expected to double by 2040 (WHO report on cancer). This is mirrored by the scientific community efforts to establish novel drug discovery protocols (Reed, 1999; Wong, 2011). Within this approach, evasion of apoptosis being a hallmark in carcinogenesis has attracted considerable interest (Pistritto et al., 2016). Given the pivotal regulatory role of the tumor suppressor protein p53 in apoptosis (Fridman and Lowe, 2003; Vazquez et al., 2008), drugging this pathway for harnessing its apoptosis-inducing functions is a rapidly growing efficient anticancer protocol (Khoo et al., 2014). p53, the so-called “guardian of the genome,” is a transcription factor that can regulate a plethora of genes controlling DNA repair, cell cycle arrest, and apoptosis (Fridman and Lowe, 2003; Vazquez et al., 2008). p53 normally induces the apoptotic cascade mainly *via* activating the pro-apoptotic proteins PMAIP1 and PUMA (Villunger et al., 2003), which are able to inhibit the mitochondrial antiapoptotic protein family such as Mcl1 and Bcl2 (Chen et al., 2005). Besides, the direct translocation of p53 to the mitochondria can induce Bcl2 family proteins and, consequently, trigger apoptosis (Mihara et al., 2003; Chipuk et al., 2004). Other findings implied that p53 can induce both caspase-dependent (Schuler et al., 2000) and -independent programmed cell death (Tovar et al., 2013) as well as cell senescence *via* the cell cycle inhibitor p21 (Lujambio et al., 2013). The transcription activity of p53 is tightly controlled by its endogenous negative regulator MDM2, which can directly conceal p53 N-terminal transactivation domain (Oliner et al., 1993) and induce its proteosomal degradation (Haupt et al., 1997). Consistent with its regulatory role, MDM2 is oncogenic when overexpressed (Ganguli et al., 2000).

In approximately 50% of human cancers, p53 is detected with mutation or deletion, whereas wild-type p53 cancers loss p53 functions due to overexpression (Eymin et al., 2002) or amplification (Oliner et al., 1992) of MDM2. Thus, targeting the interplay between p53 and MDM2 for harnessing p53 apoptosis-inducing functions has been adopted as attractive anticancer strategy. Over the last decades, active drug discovery programs have focused on validating p53–MDM2 pathway druggability. Various strategies, especially direct MDM2 inhibition, have been proposed by both academic research groups and industry (Anifowose et al., 2020; Konopleva et al., 2020). In the course of the initial hit finding, about 20 different classes of potent small-molecule MDM2 inhibitors have been introduced. Further optimization strategies disclosed about seven clinical-stage molecules (Popowicz et al., 2011; Beloglazkina et al., 2020). Of the most promising ones, for which both potency and pharmacokinetics could be optimized, are spirooxindoles.

**FIGURE 1:**
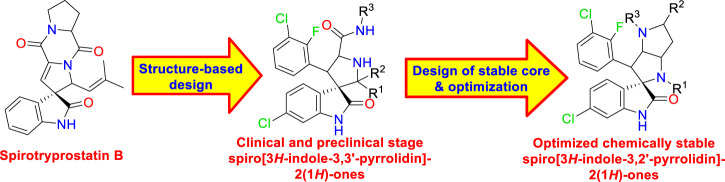
Design of spiro [3*H*-indole-3-3′-pyrrolidin]-2(1*H*)-one MDM2 inhibitors and optimization to the chemically stable spiro [3*H*-indole-3-2′-pyrrolidin]-2(1*H*)-ones series.

Spirooxindoles were first identified *via* structure-based *de novo* design as MDM2–p53 interaction inhibitors by Wang et al. (Ding et al., 2006), where the p53 Trp23 indole ring that forms a key hydrogen bond with MDM2 Leu54 and fills its hydrophobic cleft was replaced by an oxindole ring. Then additional valency utilized the oxindole C3 to install a spiro-ring inspired by natural anticancer architectures such as spirotryprostatin B. The spiro [3*H*-indole-3,3′-pyrrolidin]-2(1*H*)-ones were then introduced with suitable vectors to address the two remaining lipophilic MDM2 cavities (Leu26 and Phe19) of the p53 binding site. Consequently, several clinical-stage spirooxindoles were developed such as SAR405838 by Sanofi-Aventis (Wang et al., 2014), RO2468 by Hoffmann-La Roche (Zhang et al., 2014), and DS-3032b by Daiichi Sankyo (Nakamaru et al., 2015). However, the chirality at C2 was with epimerization in solution by reversible ring-opening retro Mannich reaction (Popowicz et al., 2010). This associated chemical instability directed modifications of Wang’s scaffold to the more chemically stable spiro [3*H*-indole-3,2′-pyrrolidin]-2(1*H*)-ones that are not prone to epimerization. With more structure-based optimization studies inspired by natural products, a fused ring system that ideally suited MDM2 was incorporated. Promising derivatives were highly selective and orally active with *in vivo* efficacy even after single-dose administration as demonstrated by BI-0252 (Figure 1) (Gollner et al., 2016).

Despite the introduction of several efficient MDM2 inhibitors, various molecular mechanisms influencing cancer cell resistance to MDM2 inhibitors were reported (Long et al., 2010; Vogelstein et al., 2013; Chapeau et al., 2017). Such resistance posed an argument about the tumor cell response to p53 activation events. Accordingly, chemotherapeutic agents that can synergize with MDM2 inhibitors, reverse resistance, and induce stronger p53 response were extensively studied. Combination studies revealed that downregulating the antiapoptotic genes, especially the Bcl2 family members, sensitizes the cancer cells to apoptosis and promotes tumor elimination (Kojima et al., 2006; Kracikova et al., 2013).

Considering the major clinical challenge represented by the tumor resistance to MDM2 inhibitors, it is reasonable that the next frontier in MDM2 research is to develop efficient inhibitors endowed with potential to modulate p53 downstream signals involved in tumor sensitization beside its intrinsic p53-activation capability. Within this context, targeting Bcl2 family attracted our interest given the evidence that inhibition of MDM2 and Bcl2 protein function synergistically induce apoptosis in cancer cells (Kojima et al., 2006; Kracikova et al., 2013).

**FIGURE 2:**
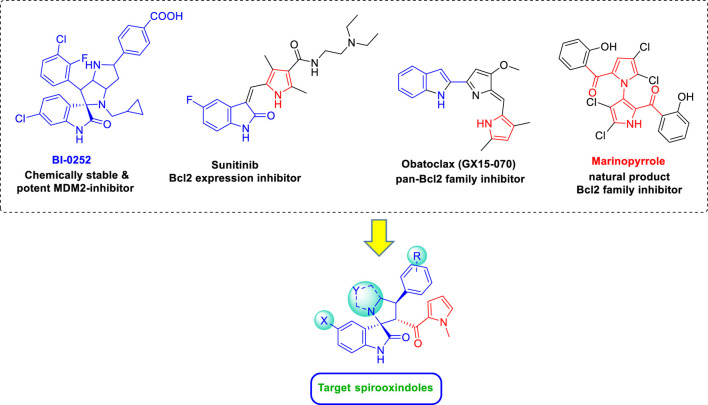
Design strategy of the target spirooxindoles.

**SCHEME 1:**
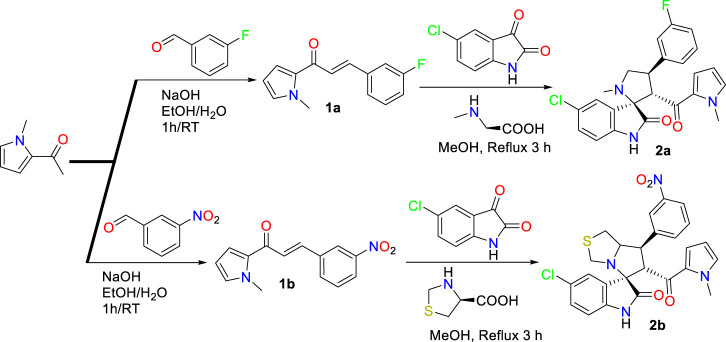
Synthesis of the spirooxindole derivatives **2a,b**.

Accordingly, in continuation to our previous work (Barakat et al., 2019; Islam et al., 2019; Islam et al., 2020), we utilized the chemically stable spiro [3*H*-indole-3,2′-pyrrolidin]-2(1*H*)-one scaffold as core for installing a pyrrole ring on the spiro ring *via* carbonyl spacer inspired by marinopyrrole; a natural marine product that disrupt Bcl2 family functions by multiple mechanisms ranging from direct inhibition to downregulation (Figure 2) (Doi et al., 2012; Wan et al., 2018). Other representative examples for pan-Bcl2 family inhibitors such as obatoclax (GX15-070) and sunitinib are supportive for our rational design study (Li et al., 2008). The spiro ring was modified as pyrrolidine and hexahydropyrrolo[1.2-*c*]thiazoline, together with the terminal aryl ring substitutions, to afford two new spirooxindoles *via* [3 + 2] cycloaddition (32CA) reaction of olefin-based *N*-methyl pyrrole with the substituted isatin, and the secondary amines Scheme 1. Recent advances made in the theoretical understanding of 32CA reactions based on the molecular electron density theory (Domingo, 2016) (MEDT) have allowed establishing a very good correlation between the electronic structure of the simplest three-atom- component (TAC) and their reactivity toward ethylene (Ríos-Gutiérrez and Domingo, 2019). The simplest AY, CH_2_–NH–CH_2_, is a very reactive *pseudodiradical* TAC participating in *pdr-type* 32CA reactions without any appreciable barrier (Domingo et al., 2010). However, the substitution on the experimental TACs stabilize them, thus, changing its electronic structure and, consequently, its reactivity to that of *zw-type* 32CA reactions (Domingo et al., 2020a). Consequently, this type of 32CA reaction demands the adequate nucleophilic/electrophilic activation of the reagents to take place (Ríos-Gutiérrez and Domingo, 2019).

The synthesized spirooxindoles were subjected to cytotoxicity screening *via* MTT assay (Mosmann, 1983; Rizk et al., 2019; Abdelmoneem et al., 2021) on normal lung fibroblasts (Wi-38) to assess their safety profiles, followed by evaluating their anticancer potencies against three selected human cancers: breast (MDA-MB 231), liver (HepG-2) and colon (Caco-2), which are reported to be among the most common leading causes of cancer death globally (WHO 2020). Flow cytometric analysis of apoptosis was then performed to test their apoptotic induction potentials, followed by immunohistochemical analysis of p53 transactivation and qRT-PCR analysis of Bcl2 gene expression. Further investigation of p53 downstream signaling status was performed *via* qRT-PCR analysis of p21 expression (Lujambio et al., 2013; Seidel et al., 2013). Finally, docking simulations of the studied spirooxindoles were conducted into MDM2 to get more information about their possible binding modes and the essential structural features.

## Results and discussion

### Chemistry

#### Synthesis

**SCHEME 2:**
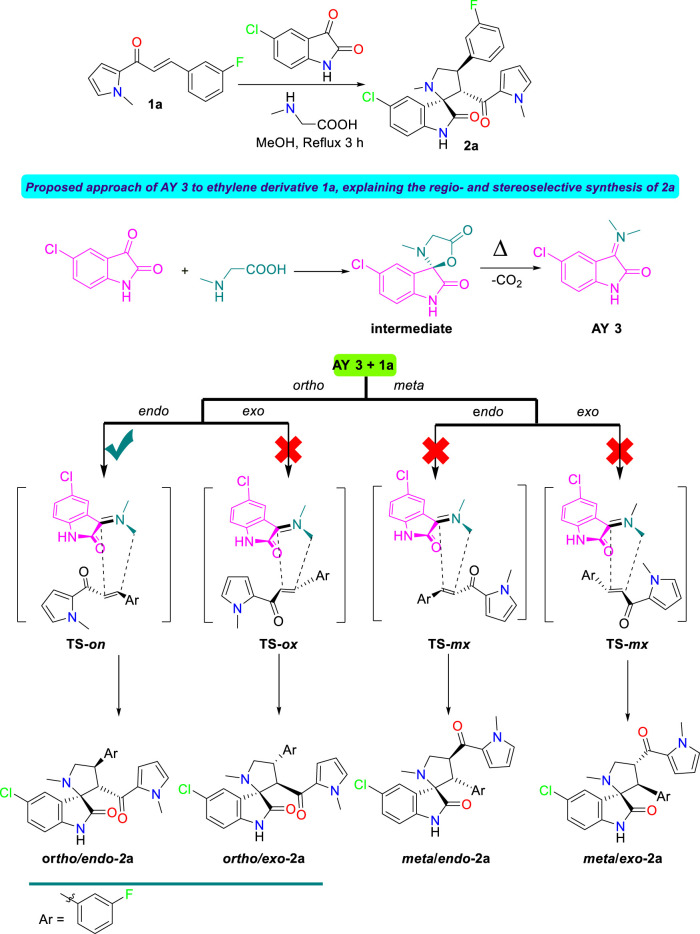
Synthesis and plausible mechanism for the desired spirooxindole derivative **2a**.

**SCHEME 3:**
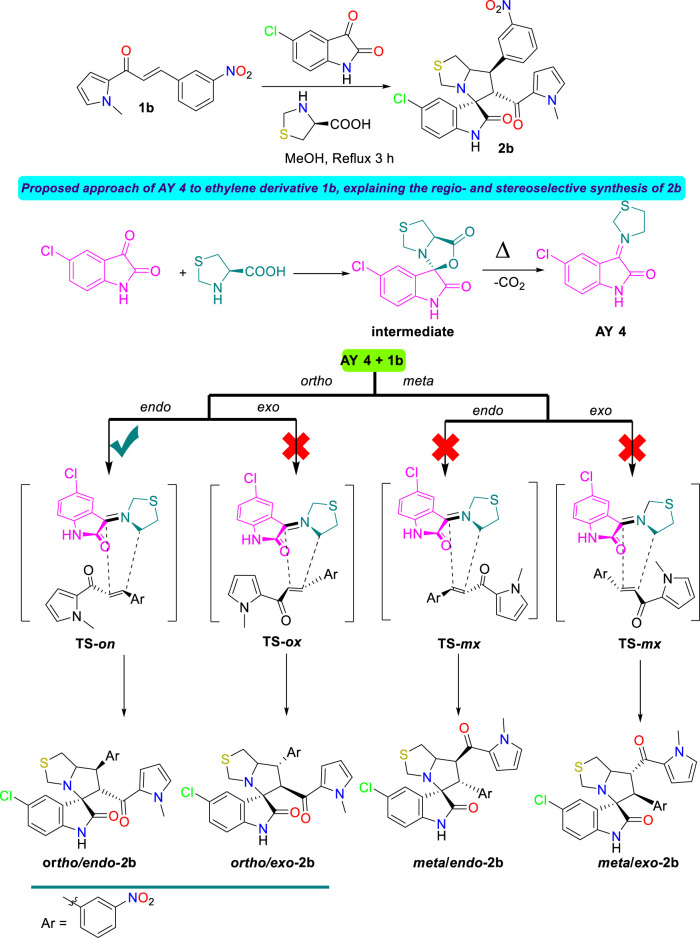
Synthesis and plausible mechanism for the desired spirooxindole derivative **2b**.

The starting material required for the synthesis of the substituted spirooxindole scaffold is the *N*-methyl pyrrole-based chalcone. The later chalcone **1a, b** is synthesized by aldol condensation of *N*-methyl-2-acetylpyrrole with the appropriate aldehydes (3-fluorobenzaldehyde/3-nitrobenzaldehyde) in the presence of aqueous NaOH, by employing a multicomponent one-pot reaction approach to furnish the requisite compound in high purity as well as regioselective and diastereoselective fashion as drawn in Schemes 2, 3. The chemical features of the substituted spirooxindole analogs were assigned based on the spectrophotometric techniques. ^1^HNMR and ^13^CNMR show all the features of the corrected protons and carbon, respectively.

#### Crystal structures of compounds **2a, b**

**FIGURE 3:**
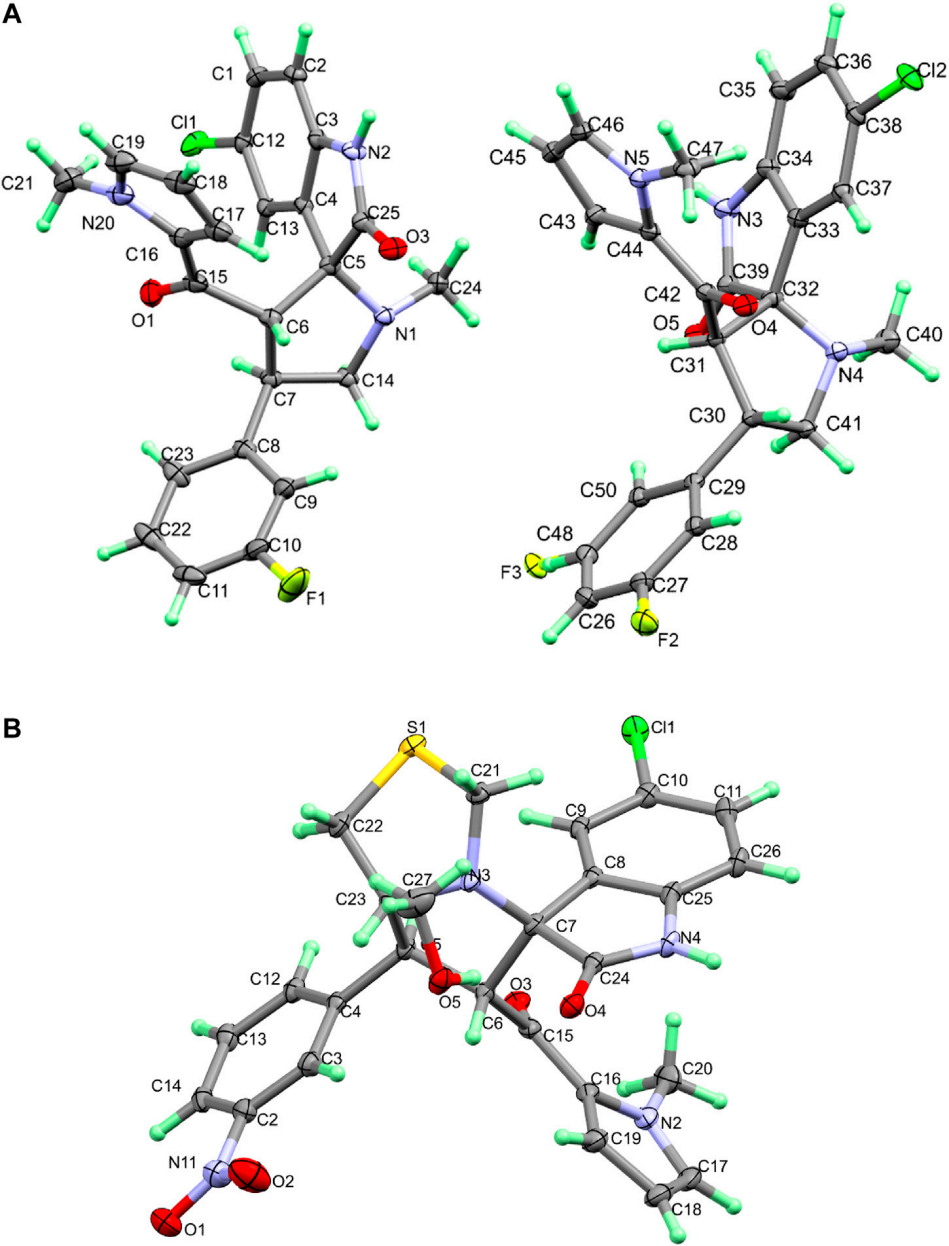
Thermal ellipsoid plots at 30% probability level for **2a (A)** and **2b (B)**.

**FIGURE 4:**
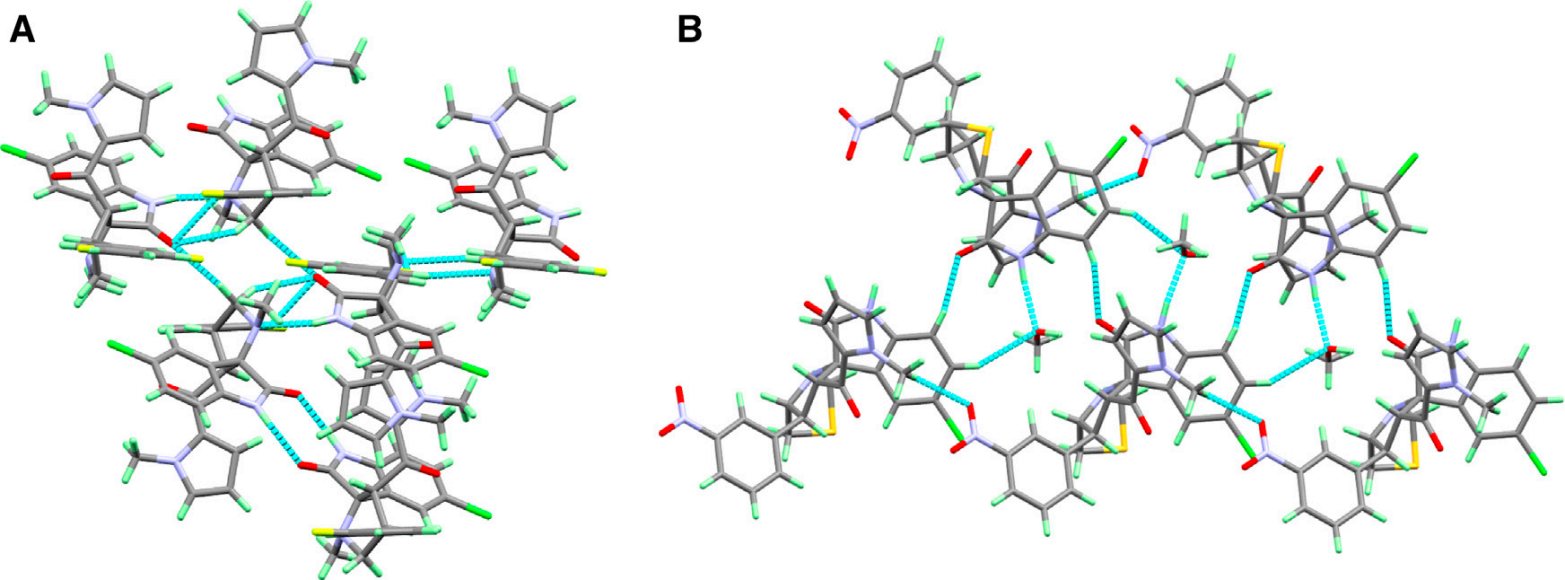
Crystal packing diagram of compounds **2a** and **2b**.

The asymmetric unit of **2a** is found to be comprised of two molecules (Figure 3A and Supplementary Table S1). Structurally, compound **2a** consists of five-membered N-methyl pyrrole (N20/C21/C16–C19) ring linked with spiroindole (C1–C7/C12–C14/C24–C25/Cl1/N1–N2/O3) moiety via the carbonyl group at C6 atom. The fluoro-substituted benzene (F1/C8–C11/C22–C23) ring was also found to be attached with spiroindole moiety at the C32 atom. One of the molecules in asymmetric unit showed 50% fluoride occupation on two carbon atoms (C27 and C48) of the ring. In the crystal lattice of compound **2a**, molecules are found to be connected *via* H2A … O3, H3A … N1, H3 … O4, H12 … O4, H15 … O4, H24 … F2, and H38 … N4 intermolecular interactions to form a three-dimensional (3D) network with donor acceptor distance of 2.9046, 3.0784, 3.2510, 3.3412, 3.4922, 3.4083, and 3.4292 Å, respectively (Figure 4 and Supplementary Table S2).

Compound **2b** crystals out as methanol solvate and was found to consist of a five-membered *N*-methyl pyrrole (N2/C16–C20) ring linked with spiroindole (C5–C11/C23–C26/Cl1/N3–N4/O4) moiety via carbonyl group at C6 atom. The spiroindole moiety was found to be further linked with S1/C21–C22 atoms forming a five-membered (N3/S1/C21–C23) ring with boat conformation. The nitro-substituted benzene (N11/O1–O2/C2–C4/C12–C14) ring was also found to be attached with spiroindole moiety at the C5 atom. The asymmetric unit also contains one methanol solvent (C27/O5) molecule (Figure 3B and Supplementary Table S1). In the three-dimensional network of crystal lattice of compound **2b**, molecules are found to be connected via H4A … O5, H1 … O5, H11 … O2, and H19 … O4 intermolecular interactions with donor acceptor distances of 2.8199, 3.4252, 3.047, and 3.2463 Å, respectively (Figure 4 and Supplementary Table S3).

#### Analysis of molecular packing

**FIGURE 5:**
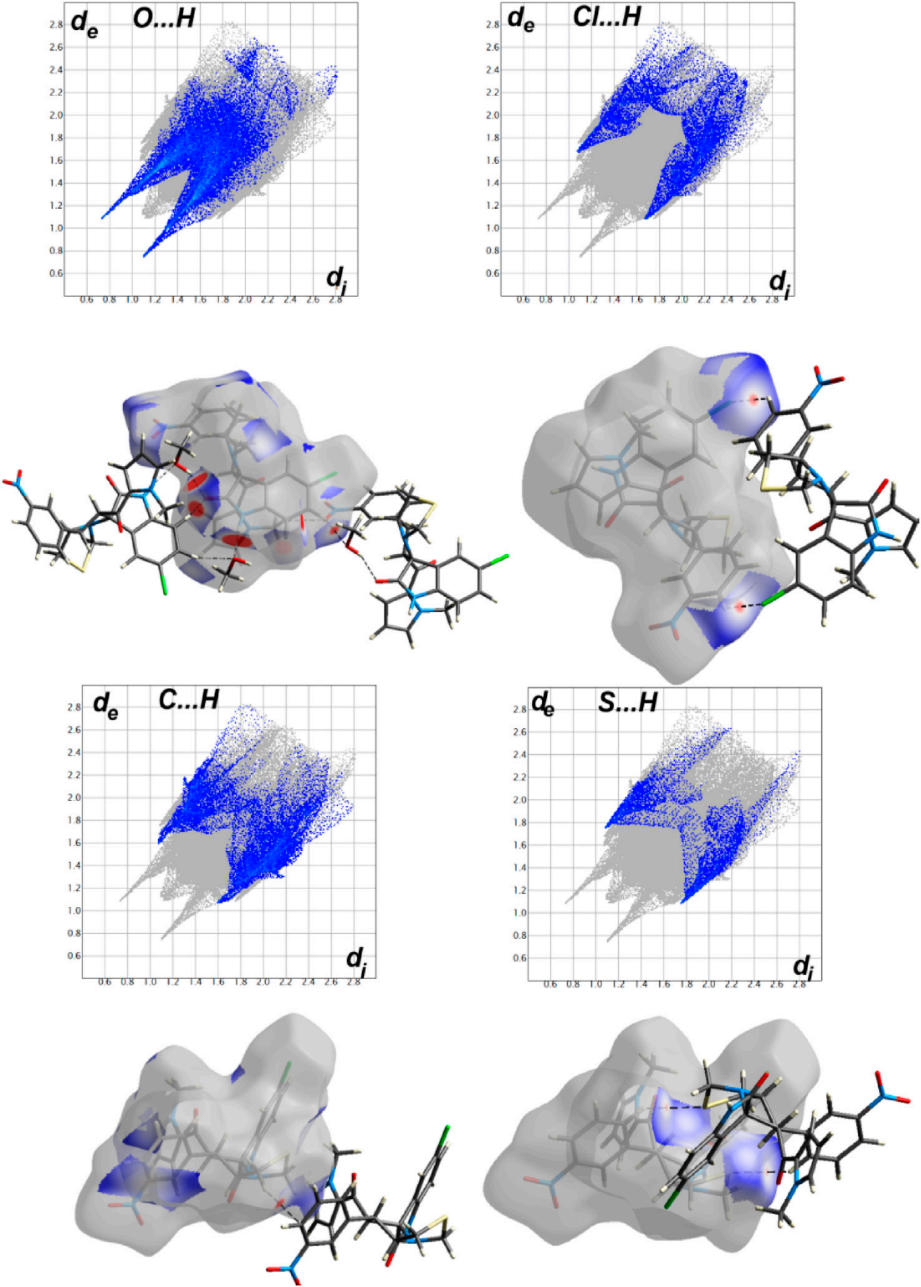
Hirshfeld analysis of **2b**.

The different contacts observed in the crystal structure of **2b** are shown in Supplementary Figure S9. The molecules are mainly packed by H … H (36.6%), O … H (23.7%), C … H (13.6%), Cl … H (9.1%), and S … H (5.6%) and N … H (3.1%) contacts. The Hirshfeld surfaces for the intermolecular interactions with contact distances shorter than van der Waals radii sum of the two elements sharing this contact are shown in Figure 5 and corresponding interaction distances are listed in Table 1. The strongest contacts are those that appeared as red spots, which correspond to O … H, Cl … H and S … H hydrogen bonds as well as C–H … π interactions.

**TABLE 1:** The short intermolecular contacts in **2b**.

Contact	Distance	Contact	Distance
O4 … H5A	1.843	O5 … H4A	1.816
O4 … H19	2.315	Cl1 … H4	2.755
O2 … H11	2.278	S1 … H3	2.842
O5 … H1	2.468	C14 … H17	2.668

**FIGURE 6:**
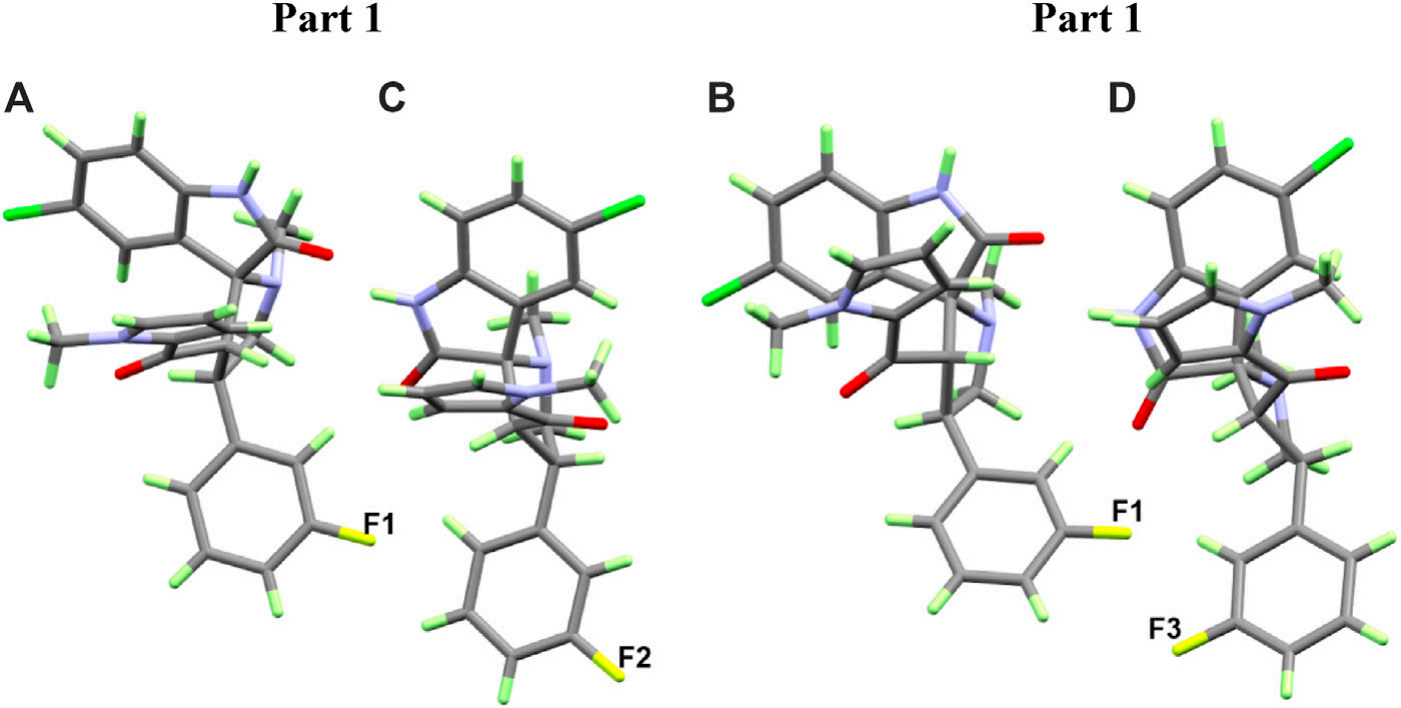
The possible orientation of the two molecular units as a consequence of the substitutional disorder in **2a**.

For **2a**, the asymmetric unit comprised two molecules of this compound. One of them showed substitutional disorder at the F2 and F3 atomic sites with H17 and H48, respectively, and with equal occupancies. As a result, we have the two possible relative orientations of the molecular units in the crystal as shown in Figure 6. Presentation of all possible contacts and their percentages for the four possible in this crystal structure are shown in Supplementary Figure S10.

**FIGURE 7:**
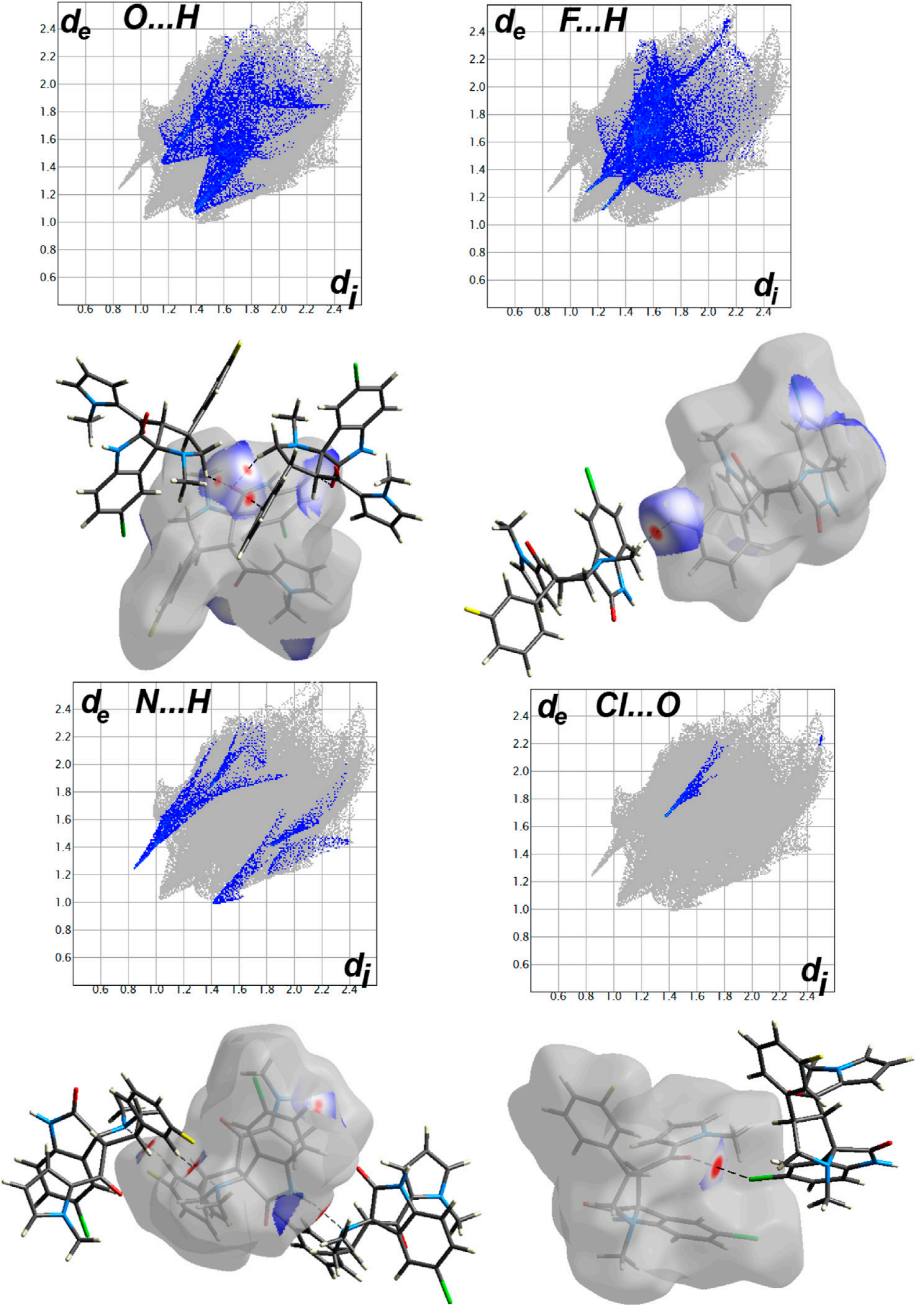
Hirshfeld analysis of **2a**.

In this regard, we have four sets of results for this compound as shown in Supplementary Figure S10. The most important contacts in **2a** are H … H (42.2–46.4%), C … H (16.1–19.8%), F … H (6–10.9%), Cl … H (8.1–12.1%), and O … H (8.0–11.6%). The Hirshfeld analysis of the shortest contacts observed in case of **2a** is collected in Figure 7 and a list of these interactions, along with the contact distances, is given in Table 2. It is worth noting that some short halogen-bonding interaction with contact distance of 3.042 Å for Cl1 … O5 contact was noted in this compound. It is important to mention that the rotation of the fluorophenyl ring around the C–C bond, which connects it with the spiro system leads to some weak F … F contacts only found in part 2 of the disordered system. The percentage of the F … F interactions does not exceed 1.1% from the whole intermolecular contacts.

**TABLE 2:** The short intermolecular contacts in **2a**.

Contact	Distance	Contact	Distance
O3 … H2A	1.913	N1 … H3A	2.085
O3 … H41	2.587	N4 … H38	2.406
O4 … H12	2.467	Cl1 … O5	3.042
O4 … H15	2.505	F2 … H24	2.349
O4 … H3	2.51		

#### DFT structural studies

Based on the x-ray structure model, compound **2a** could exist in two possible conformers as shown in Supplementary Figure S13. The optimized molecular geometries of both conformers were calculated and presented in comparison with the x-ray structure model in Supplementary Figure S13. The optimized and calculated bond distances are in good agreement (Supplementary Table S4; Supplementary Data). There are good correlations between the calculated and experimental bond distances for both conformers (Supplementary Figure S14). The correlation coefficients are 0.9863 and 0.9916, respectively. It is worth to note that the total energies of 2a_F1 and 2a_F2 are −1,130,708.937 and −1,130,711.000 eV, respectively, with an energy difference of 2.063 eV. The energy difference is very small, which confirms the coexistence of both conformers as revealed from the x-ray structure analysis. Also, the dipole moments of the two conformers are close to each other. The calculated dipole moments values are 4.1294 and 4.6453 Debye for 2a_F1 and 2a_F2, respectively.

On the other hand, the optimized molecular geometries of **2b** with and without the crystallized methanol molecule are given in Supplementary Figure S15. Also, a comparison between the calculated and optimized geometries is presented in the lower part of this figure together with the correlations between the calculated and experimental bond distance for both molecules. The correlations coefficients are high (0.9951–0.9955) indicating the good agreement between the calculated and x-ray structures (Supplementary Table S5; Supplementary Data). The calculated dipole moment is predicted to be higher in presence of the crystallized solvent (6.9043 Debye) compared with 5.8779 Debye for the compound without the crystallized methanol molecule.

**FIGURE 8:**
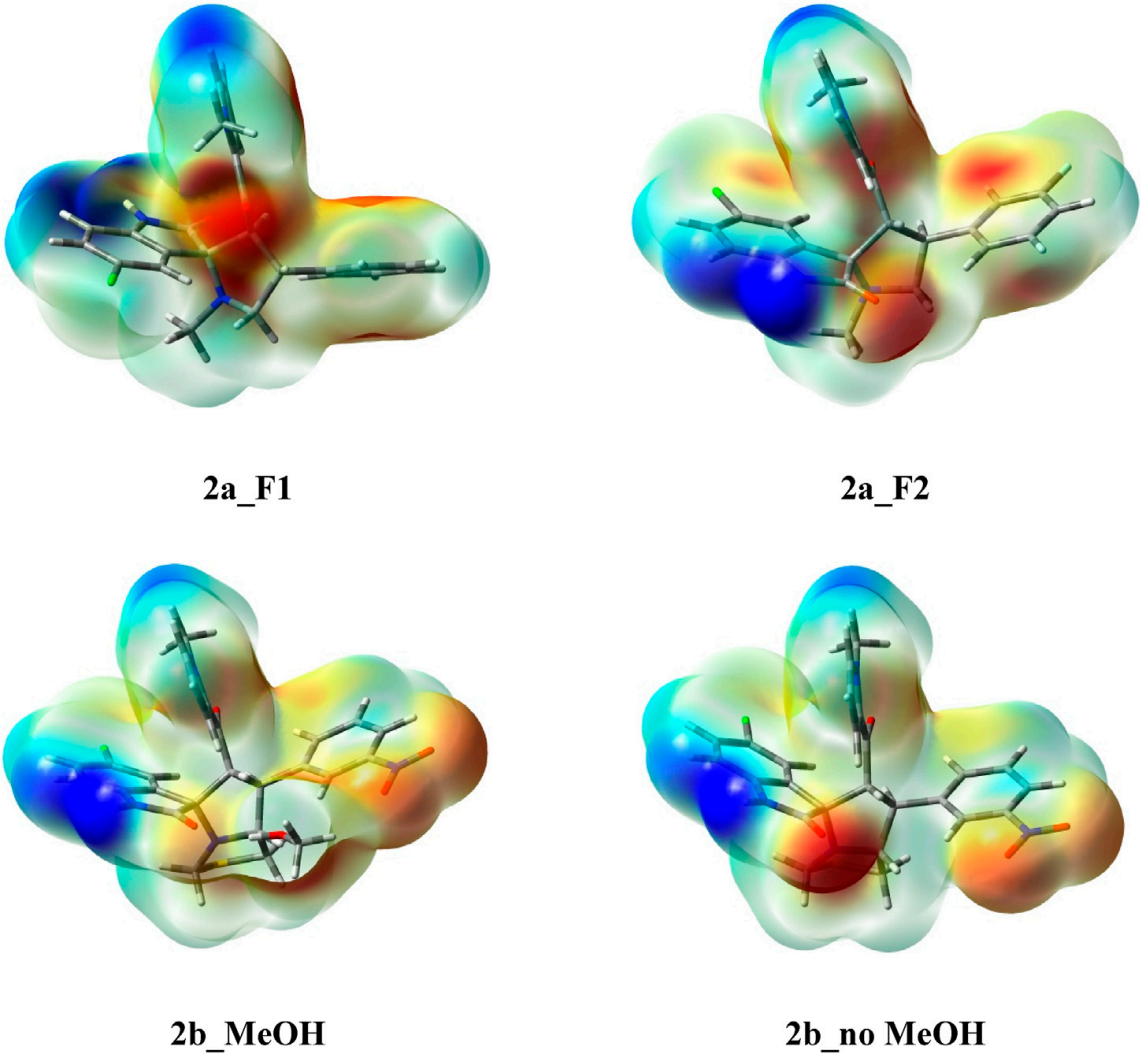
The molecular electrostatic potential (MEP) maps of the studied compounds. The color index from blue to turquoise to yellow to red indicat the more negative electron density.

The molecular electrostatic potential (MEP) map is a colored presentation that indicates the different charged regions in molecular systems. Red-colored regions are the most negative, while the most positive regions have a blue color (Figure 8). These regions represent the most proper regions for hydrogen-bonding interactions as hydrogen bond acceptor and hydrogen bond donor, respectively. The red regions are related to the oxygen atoms in both compounds which represent the most suitable regions for hydrogen bonding interactions as hydrogen bond acceptor sites. In contrast, the blue regions are related to the NH proton, which acts as a hydrogen bond donor. These results are in good agreement with the x-ray structure of the studied compounds.

The natural charges at the different atomic sites are listed in Supplementary Table S5; (Supplementary Data). In **2a**, the most negative atomic sites are the oxygen atoms of the C=O groups and the nitrogen sites of the spiro-system. The calculated charges are in the range of −0.597 to −0.616 and −0.512 to −0.611, respectively. On other hand, the most positive atomic sites are the NH proton and the carbonyl carbon atoms with natural charges ranging from 0.408 to 0.410 e and 0.541 to 0.727 e, respectively. Similarly, for **2b**, the two carbonyl oxygen atoms are the most negative with natural charges ranging from 0.598 to −0.641 e while the corresponding carbon atoms and the NH proton are the most positive sites with natural charges in the range of 0.536–0.742 and 0.410–0.412 e, respectively. In addition, the nitrogen atom of the nitro group has also a high positive charge of 0.517–0.519 e.

#### Conceptual DFT analysis of the 32CA reactions of AYs 3 and 4 with ethylene derivatives **1a** and **1b**

The reactivity indices defined within the conceptual DFT (CDFT) (Parr and Yang, 1989; Domingo et al., 2016) have shown to be powerful tools to understand the reactivity in polar reactions. The global reactivity indices, namely, the electronic chemical potential *μ*, chemical hardness *η*, global electrophilicity ω, and global nucleophilicity *N*, for the reagents involved in these 32CA reactions are gathered in Table 3.

**TABLE 3:** B3LYP/6-31G(d) reactivity indices, in eV, of AYs **3** and **4**, and ethylene derivatives **1a** and **1b**.

	*μ*	*η*	ω	*N*
Ethylene **1b**	−4.37	3.64	2.62	2.94
Ethylene **1a**	−3.97	4.04	1.96	3.13
AY **4**	−3.34	3.29	1.70	4.13
AY **3**	−3.26	3.34	1.60	4.19

The electronic chemical potentials (Parr and Yang, 1989) *μ* of AYs, −3.26 (**3**) and −3.34 (**4**) eV, are higher than those of the ethylene derivatives, −3.97 (**1a**) and −4.37 (**1b**) eV, indicating that along a polar 32CA reaction, the global electron density transfer (GEDT) (Domingo, 2014) will take place from these AYs to the ethylene derivatives, the reactions being classified as the forward electron density flux (FEDF) (Domingo et al., 2020b).

AYs **3** and **4** have an electrophilicity ω index (Parr et al., 1999) of 1.60 (**3**) and 1.70 (**4**) eV, respectively, being classified as strong electrophiles within the electrophilicity scale (Domingo et al., 2016). On the other hand, they have a nucleophilicity *N* index (Domingo et al., 2008a) of 4.19 (**3**) and 4.13 (**4**) eV, being classified as strong nucleophiles within the electrophilicity scale (Domingo et al., 2016). The strong nucleophilic character of these AYs, higher than 4.0 eV, allows their classifications as a supernucleophile (Chamorro et al., 2020).

Ethylene derivatives **1a** and **1b** have an electrophilicity ω index of 1.96 (**1a**) and 2.62 (**1b**) eV, respectively, being classified as strong electrophiles. On the other hand, they have a nucleophilicity *N* index of 3.13 (**1a**) and 2.94 (**1b**) eV, being classified as a strong nucleophile and in the borderline of moderate nucleophiles, respectively. The presence of a strong electron-withdrawing NO_2_ group in ethylene **1b** increases the electrophilicity and decreases the nucleophilicity of this species with respect to those of ethylene **1a**. The supernucleophilic character of AYs **3** and **4** together with the strong electrophilic character of ethylenes **1a** and **1b** indicate that the corresponding 32CA reactions will have polar character, being classified as FEDF (Domingo et al., 2020b).

#### Study of the reaction mechanism

**SCHEME 4:**
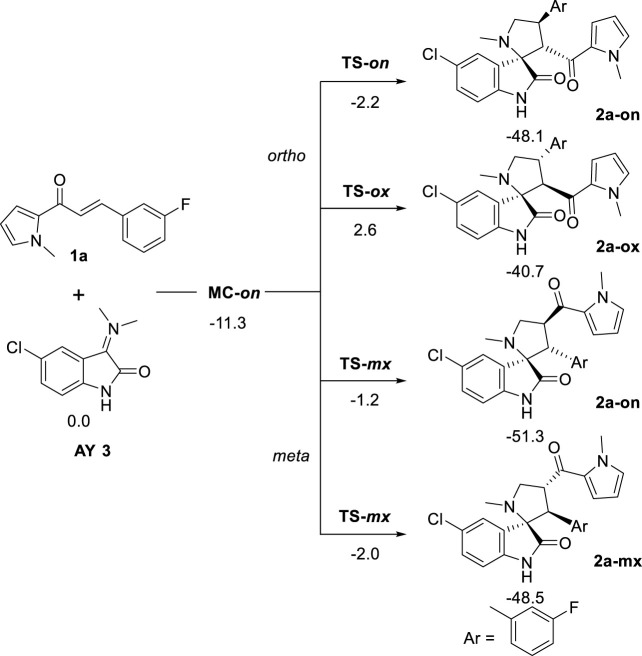
32CA reaction of AY **3** with the ethylene derivative **1a**. *ω*B97X-D/6-311G(d,p) relative energies in methanol, with respect to separated reagents, are given in kcal·mol^−1^

The mechanism of the 32CA reaction of AY **3** with ethylene **1a** was theoretically studied. Due to the nonsymmetry of both reagents, two pairs of *endo* and *exo* stereoisomeric and two pairs of *ortho* and *meta* regioisomeric reaction paths are feasible. The four competitive reaction paths were studied (see Scheme 4). Analysis of the stationary points found in these reaction paths indicates that this 32CA reaction takes place though a one-step mechanism. The *ω*B97X-D/6-311G(d,p) relative energies in methanol are given in Scheme 4.

A series of molecular complexes (MCs) in which the two reagents are already joined by weak intermolecular interactions were also found. Only the most stable of them, **MC-on**, was selected as the energy reference. The distance between the two frameworks at this MC is ca. 3.2 Å; **MC-on** is found 11.3 kcal·mol^−1^ below the separated reagents (see Scheme 4). The most favorable **TS-on** is found 2.2 kcal·mol^−1^ below the separated reagents, the corresponding reaction path being strongly exothermic by 48.1 kcal·mol^−1^. Some appealing conclusions can be obtained from the relative energies given in Scheme 4: 1) **TS-on** is found below the separated reagents, but if the formation of **MC-on** is considered, the activation energy becomes positive by 9.1 kcal·mol^−1^. 2) The regioisomeric **TS-mx** is found only 0.2 kcal·mol^−1^ above **TS-on**. These energy results do not account for the only formation of **2-on** *via* **TS-on**. 3) This 32CA reaction is totally *endo* stereoselective as **TS-ox** is found 4.8 kcal·mol^−1^ above **TS-on**. 4) The high exothermic character of the formation of **2a-on**, −48.1 kcal mol^−1^, makes this 32CA reaction irreversible. Consequently, cycloadduct **2a-on** is formed by kinetic control.

**FIGURE 9:**
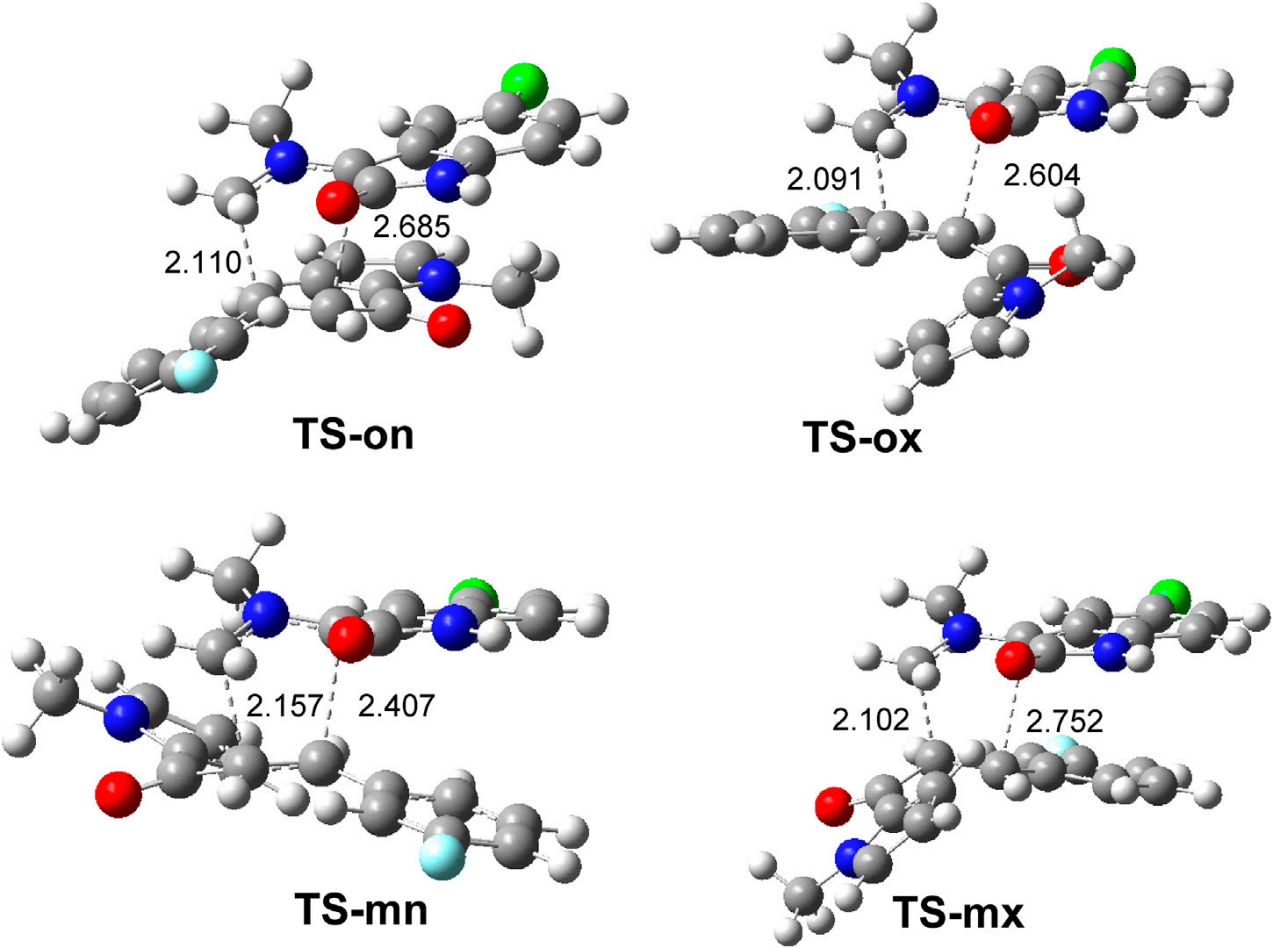
*ω*B97X-D/6-311G(d,p) geometry of TSs in methanol. The distances are given in Angstrom.

The geometries of the four TSs are given in Figure 9. The C−C distances between the four interacting carbons at the four TSs indicates that they correspond with asynchronous C−C single bond formation processes, in which the shorter C−C distance corresponds to that involving the methylene CH_2_ carbon of AY **3**. At the most favorable **TS-on**, the C−C distances between the two pairs of interacting carbons, 2.110 and 2.685 Å, indicate that this TS is associated with a high asynchronous C−C single bond formation process, in which the shorter C−C distance corresponds to that involving also the most electrophilic *ß*-conjugated carbon of the ethylene derivative **1a**. Analysis of the intrinsic reaction coordinates (Fukui, 1970) associated to high asynchronous **TS-on** indicates that this 32CA reaction takes place through a nonconcerted *two-stage one-step* mechanism (Domingo et al., 2008b) in which the formation of the second C−C single bond begins when the first C−C single bond is completely formed.

Finally, analysis of GEDT (Domingo, 2014) at TSs permits assessment of the polar character of this 32CA reaction. GEDT values lower than 0.05 e correspond with nonpolar processes, while values higher than 0.20 e correspond with polar processes. The GEDT values at the TSs are 0.19 e (**TS-on**), 0.13 e (**TS-oxn**), 0.21 e (**TS-mn**), and 0.22 e (**TS-mx**). These values, which are a consequence of the supernucleophilic character of AY **3** and the strong nucleophilic character of ethylene **1a**, indicate that this 32CA reaction has a polar character. The flux of the electron density, which goes form AY **3** to ethylene **1a**, classifies this 32CA reaction as FEDF, in clear agreement with the analysis of the CDFT indices.

### Biological evaluation

#### Cytotoxicity screening

**FIGURE 10:**
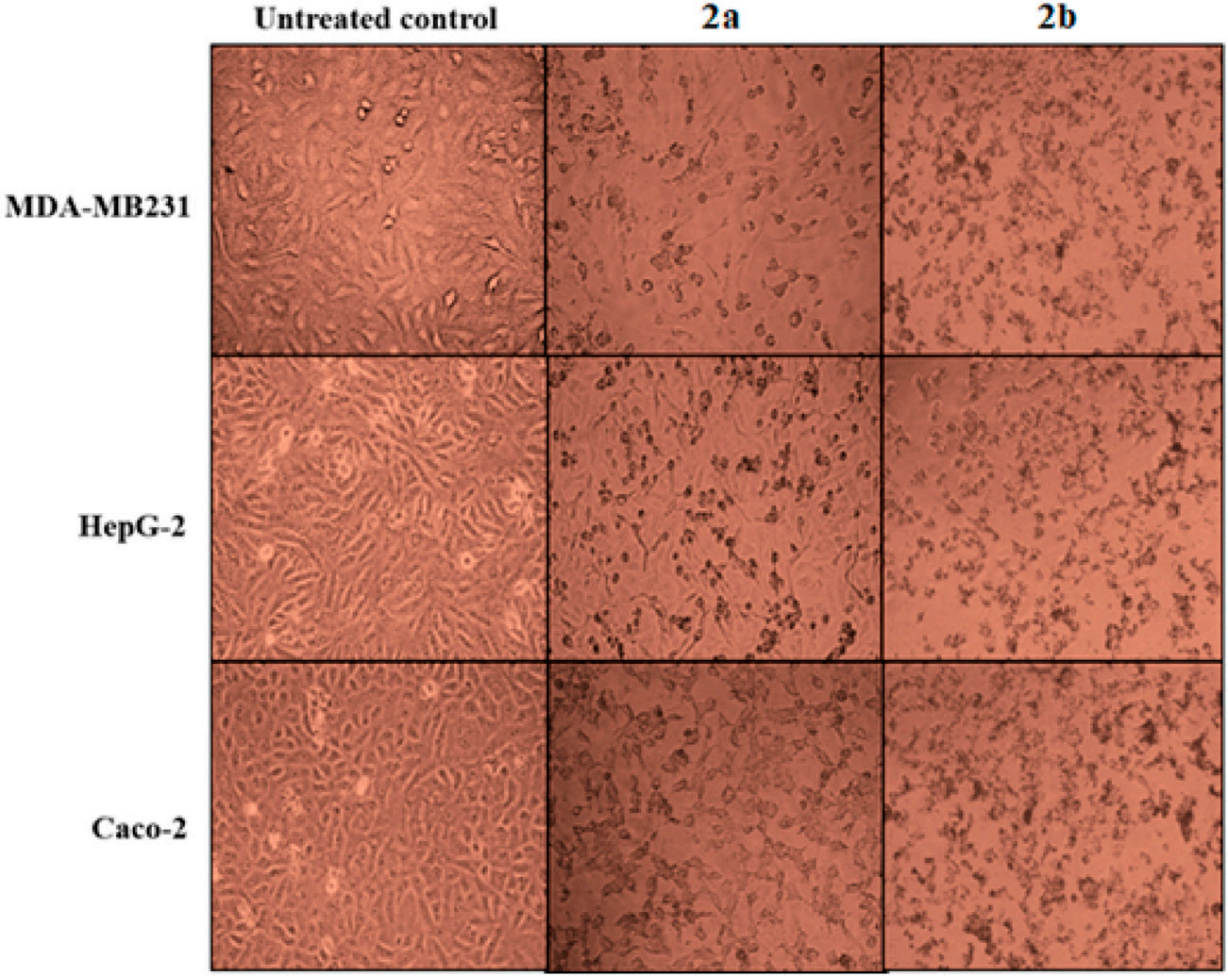
Morphological alterations of **2a-** and **2b**-treated MDA-MB 231, HepG-2, and Caco-2 cells.

The studied spirooxindoles (**2a** and **2b**) were screened for possible cytotoxic activities on normal lung fibroblasts (Wi-38) for assessment of their safety profiles. Then they were evaluated for their anticancer potential against three selected cancer cell lines (MDA-MB 231, HepG-2, and Caco-2) compared with 5-fluorouracil (5-FU) via MTT assay (Table 4) (Mosmann, 1983; Rizk et al., 2019; Abdelmoneem et al., 2021). Compound **2b** was superior to 5-FU and **2a** against the screened cells within its safe dose (EC_100_). Obviously, it recorded single-digit nanomolar IC_50_ values against MDA-MB 231 and Caco-2 cells. These pronounced anticancer activities were observed as severe shrinkage of the treated cancer cells (Figure 10) (Jerabek-Willemsen et al., 2011; Seidel et al., 2013). Compound **2a** was also more potent than 5-FU against MDA-MB 231; however, it exhibited its anticancer effect beyond its respective EC_100_; hence, it lacked considerable selectivity. Unfortunately, it lacked potency HepG-2 and Caco-2 cells.

**TABLE 4:** Cytotoxicity of the spirooxindoles **2a** and **2b**.

Compound no	EC_100_ (µM) Wi-38	IC_50_ (µM)		
		MDA-MB 231	HepG-2	Caco-2
**2a**	0.1166 ± 0.0159	0.7424 ± 0.0597	33.5660 ± 4.5720	6.2330 ± 1.4650
**2b**	0.2269 ± 0.0124	0.0018 ± 0.0004	0.0569 ± 0.0020	0.0028 ± 0.0020
5-FU	0.6013 ± 0.0810	7.0500 ± 0.2040	4.8290 ± 0.2960	1.0480 ± 0.1560

Note. *Values are presented as mean ± SEM.

#### Flow cytometric annexin V/propidium iodide analysis of apoptosis

**FIGURE 11:**
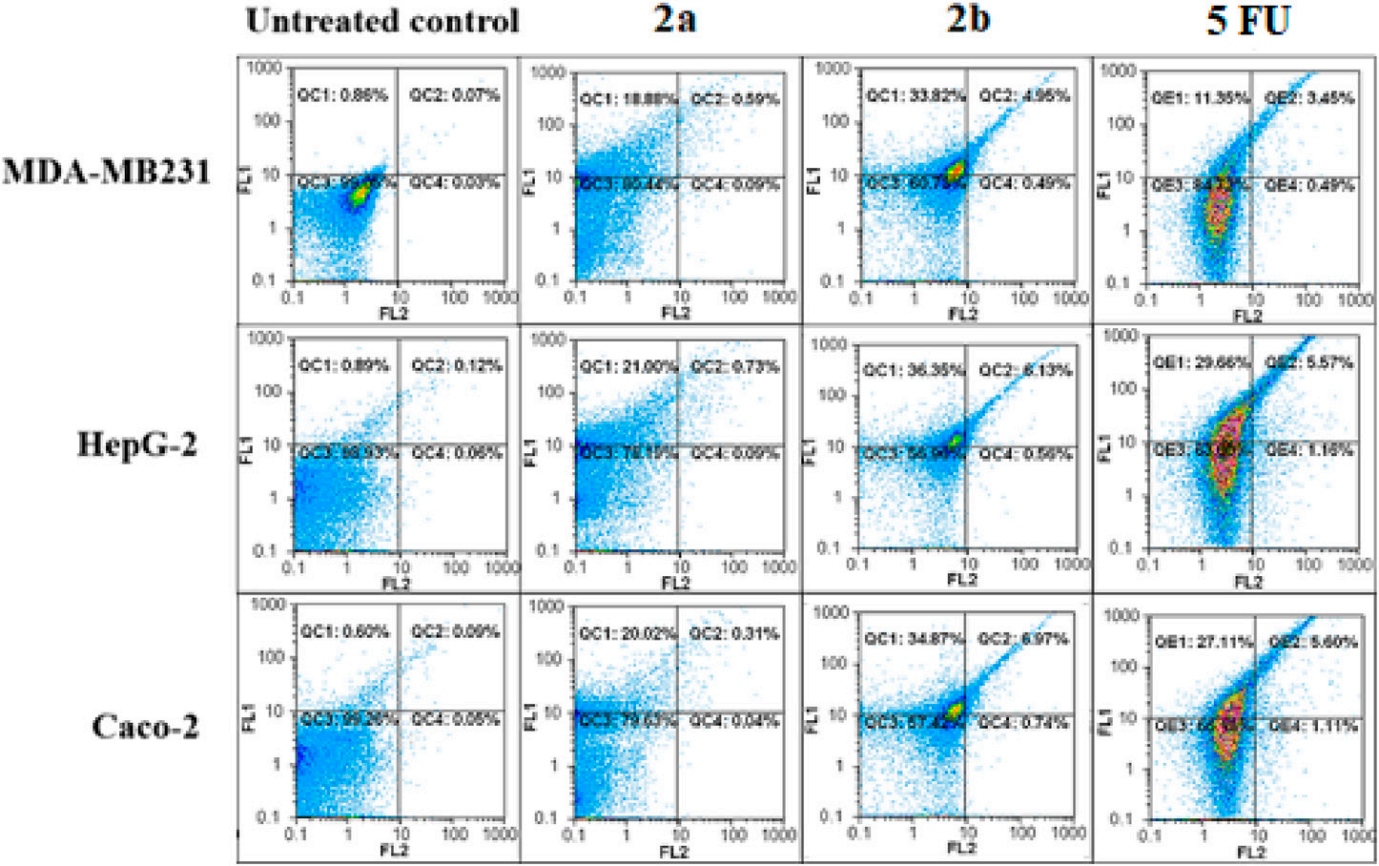
Flow charts of annexin-PI analysis of **2a-** and **2b**-treated MDA-MB 231, HepG-2, and Caco-2 cells compared with 5-fluorouracil (5-FU)-treated cells.

The studied derivatives were evaluated for their apoptotic induction potential by flow cytometric analysis (Seidel et al., 2013). As illustrated (Figure 11; Table 5 **)**, **2b** demonstrated the highest apoptosis-dependent anticancer activity (>39%) in MDA-MB 231, HepG-2, and Caco-2 cells. The apoptotic effect of **2b** was higher than that of the reference drug (13.02%–34.95%) in three studied cancer cell lines. Meanwhile, **2a** recorded relatively lower apoptotic population percentages than **2b** and 5-FU in HepG-2 and Caco-2 cells (Table 5). In MDA-MB 231 cells, **2a** exhibited significantly higher apoptotic activity than 5-FU. The results were consistent with that of the MTT assay.

**TABLE 5:** Apoptotic cell population percentages in **2a-** and **2b-**treated MDA-MB 231, HepG-2, and Caco-2 cells.

Compound no	Total % of apoptotic population		
	MDA-MB 231	HepG-2	Caco-2
Untreated cells	0.835 ± 0.1^d^	0.605 ± 0.09^d^	0.99 ± 0.02^d^
**2a**	21.805 ± 2.335^b^	23.48 ± 1.750^c^	23.320 ± 2.99^c^
**2b**	39.44 ± 0.67^a^	43.075 ± 0.6^a^	42.095 ± 0.25^a^
6-FU	13.02 ± 1.78^c^	31.59 ± 1.12^b^	34.95 ± 0.28^b^

Note. *Values are presented as mean ± SEM. Different letters are significantly different in the same column at *p* < 0.05.

#### Immunohistochemical analysis of p53 protein overexpression

**FIGURE 12:**
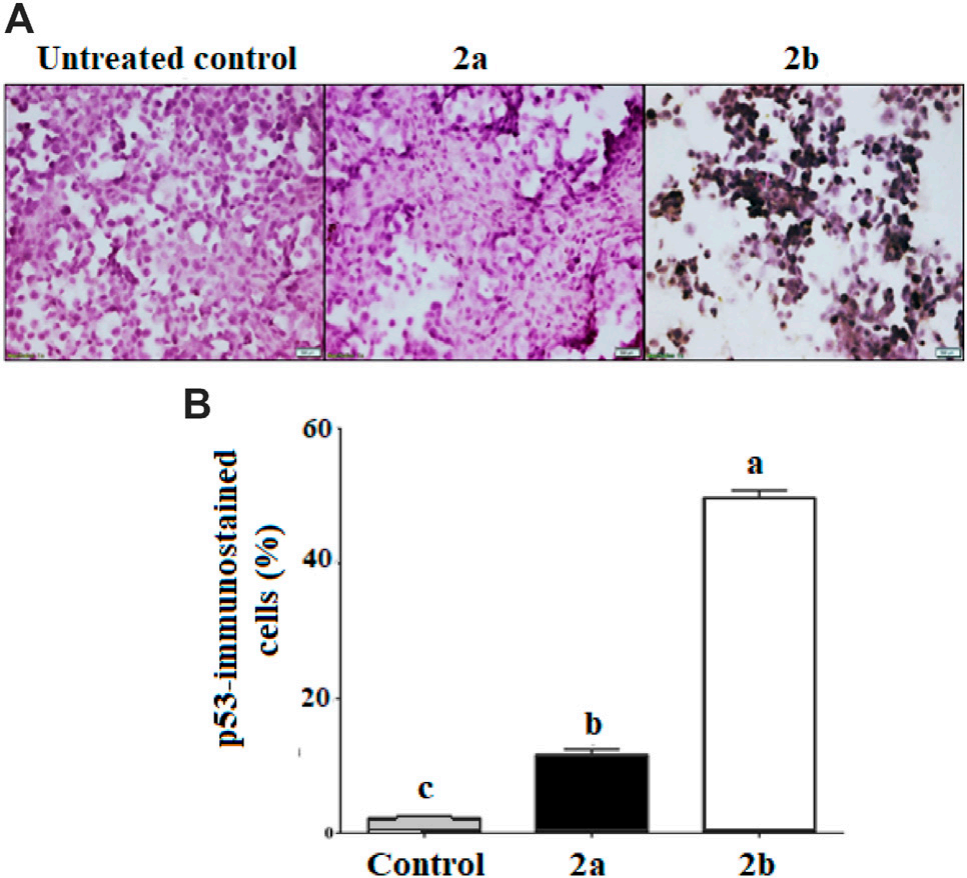
p53 immunohistochemical analysis of p53 expression in **2a-** and **2b**-treated HepG-2 cells. **(A)** Immunostaining images of p53-treated and untreated cells (magnification ×400) with **(B)** representative percentages of the positive immunostaining HepG-2 cells. Different letters are significantly different at *p* < 0.05.

We analyzed p53 immunoreactivity in HepG-2 cells treated with the two studied spirooxindoles, **2a** and **2b**. Interestingly, after exposure of HepG-2 cells to **2a** and **2b** for 72 h, a significant increase was detected in p53-positive cells. Figure 12 shows that p53 transactivation in the **2b-**treated HepG-2 cells (47.24%) was significantly higher than that in the **2a**-treated ones (9.12%).

#### qRT-PCR analysis of Bcl2 gene expression

**FIGURE 13:**
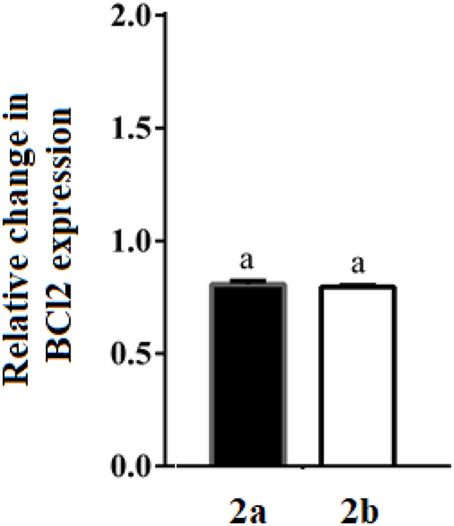
Change in gene expression of Bcl2 in the treated HepG-2 cells. Different letters are significantly different at *p* < 0.05.

RT-PCR analysis of Bcl2 gene in HepG-2 cells after treatment with the studied spirooxindoles revealed a 1.25-fold downregulation relative to the untreated cells (Figure 13). Both compounds were nearly equipotent.

#### qRT-PCR analysis of p21 gene expression

**FIGURE 14:**
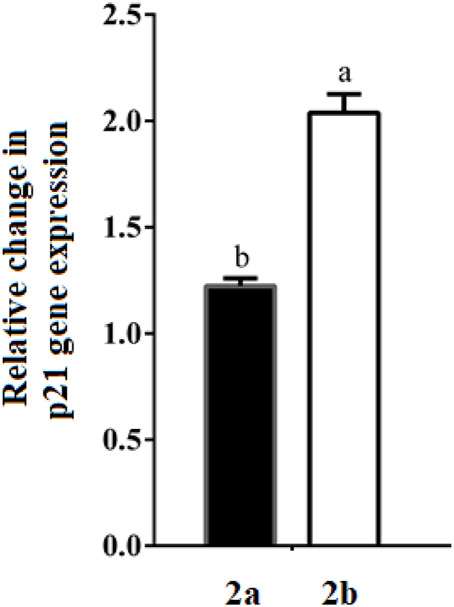
Change in gene expression of p21 in the treated HepG-2 cells. Different letters are significantly different at *p* < 0.05.

P53 mediates its DNA damage-induced checkpoint *via* transactivation of a plethora of apoptotic genes. Of these, p21 mediates G1 growth arrest (Alexander et al., 2014). Given its main p53-dependent antiproliferative role, p21 gene expression level was quantified by qRT-PCR in HepG-2 cells treated with the studied compounds (Figure 14). Herein, **2b** upregulated p21 expression by two-folds, whereas, **2a** recorded a 1.22-fold increase. This observation may be subsequent to p53-transactivation by the studied spirooxindoles.

### Molecular modeling

#### Docking simulations

**FIGURE 15:**
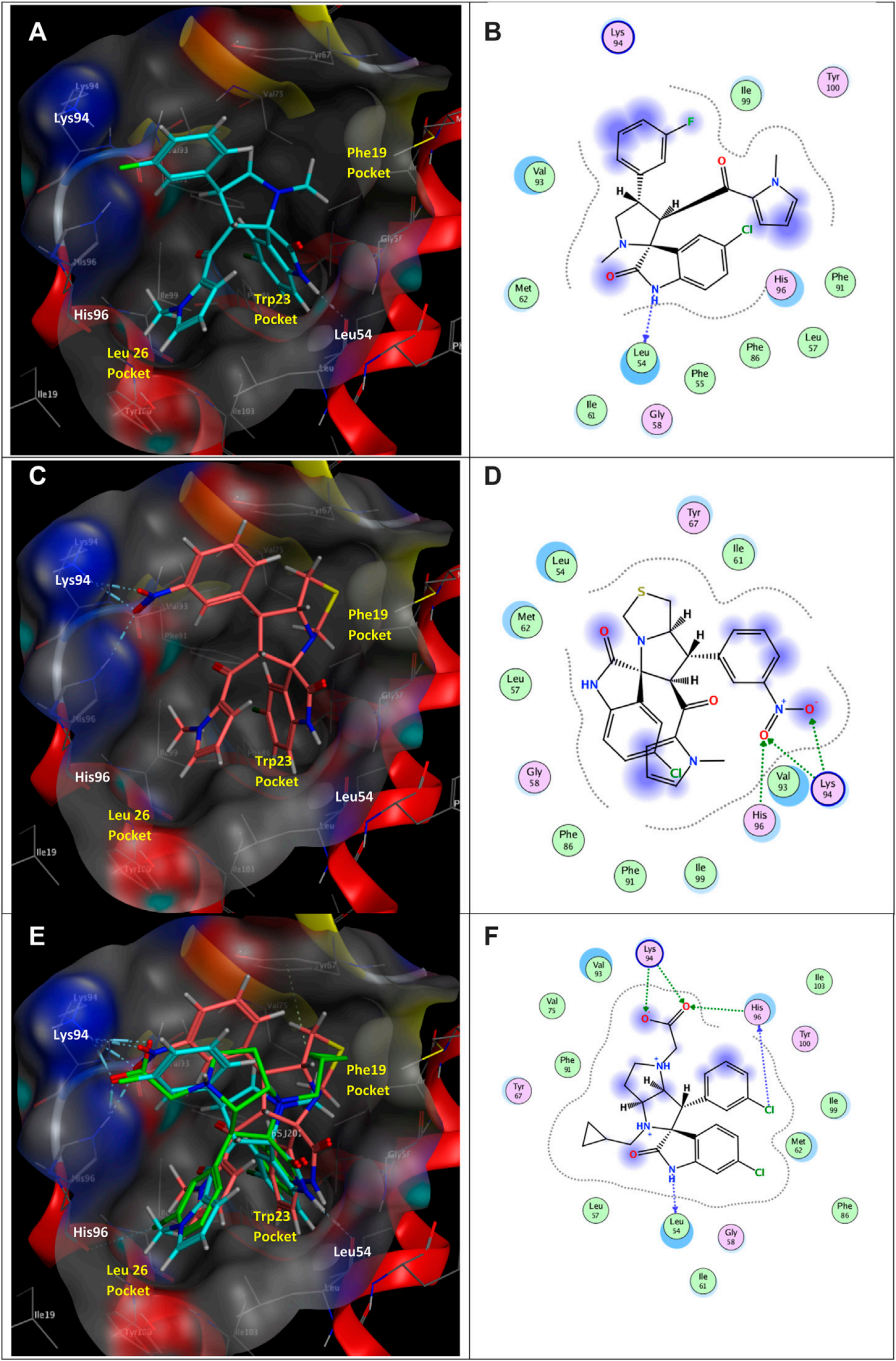
**(A)** Three-dimensional (3D) binding mode of **2a** (cyan sticks), **(B)** 2D binding mode of **2a**, **(C)** 3D binding mode of **2b** (dark pink sticks), **(D)** 2D binding mode of **2b**, **(E)** overlay of **2a** and **2b** with the co-crystalized human homolog of mouse double minute 2 (MDM2) inhibitor (green sticks), **(F)** 2D binding mode of the co-crystalized ligand 6SJ in MDM2 (PBD ID: 5LAW) (Gollner et al., 2016). The Leu26, Trp23, and Phe19 pockets of p53 are indicated as well as the key hydrogen bond interactions with MDM2 Leu54.

The coordinates of MDM2 co-crystallized with the spiro [3*H*-indole-3,2′-pyrrolidin]-2(1*H*)-one inhibitor 6SJ were downloaded from the protein data bank (PBD ID: 5LAW) (Gollner et al., 2016) and prepared by MOE 2016.0802 “QuickPrep” module (Muegge et al., 2011). The studied spirooxindoles **2a** and **2b** were built, energy minimized, then docked into the binding site of the inhibitor. The best binding modes (Figure 15) showed that the indolinone ring of both compounds were buried into the Trp23_(p53)_ pocket. However, only **2a** formed the key hydrogen bond interactions with the backbone Leu54_(MDM2)_ as the reference inhibitor 6SJ. Interestingly, the nitro group of **2b** offered hydrogen bonding interactions with His96_(MDM2)_ in the Leu26_(p53)_ pocket and Lys94_(MDM2)_. In light of these results, it may be assumed that the studied derivatives can share some key interactions with the reference MDM2 inhibitor.

#### *In silico* Prediction of Physicochemical Properties, ADME and Drug-Likeness

Physicochemical properties, ADME, and drug-likeness parameters are extensively employed as lead identification tools. Herein, *SwissADME* (Daina et al., 2017) was utilized to predict the important physicochemical properties formulating the drug-likeness parameters of the studied spirooxindoles **2a** and **2b** (Table 6). Both compounds recorded drug-like bioavailability according to the parameters of Lipinski (Lipinski et al., 2017), Veber (Veber et al., 2002), and Muegge (Muegge et al., 2011). Compound **2a** showed full accordance, whereas **2b** exhibited only single Lipiniski violation regarding its molecular weight. PreADMET 2020 (https://preadmet.bmdrc.kr/adme/) (accessed August 14, 2020) was also employed for ADME prediction. The studied compounds displayed acceptable predicted aqueous solubility; however, **2a** recorded relatively better solubility than **2b**. Both were predicted to possess excellent intestinal absorption (>96%). Compound **2a** displayed medium BBB penetration, whereas **2b** was poorly absorbed by the CNS, thus predicted to be devoid of CNS-related side effects. Both recorded moderate predicted permeability through the Caco-2 cells model. Compound **2a** was moderately absorbed by MDCK, unlike the relatively poorly absorbed **2b**. The predicted plasma protein binding profile of **2b** was almost 100%. Compound **2b** recorded medium plasma protein binding (88%), thus it was expected to be more available for transport across various membranes than **2a**. Both compounds were predicted to inhibit cytochromes CYP3A4 and CYP2D6. They showed no detected PAINs indicating genuine activities.

**TABLE 6:** *In silico* predicted physicochemical properties, ADMET, and drug likeness of the spirooxindoline derivatives **2a** and **2b**.

Cpd. No	Physiochemical parameters							ADME							Bioavailability and drug likeness			
	LogP^a^	M.Wt^b^	HBA^c^	HBD^d^	NROTB^e^	TPSA^f^	S^g^	HIA^h^	PPB^i^	BBB^j^	Caco2^k^	MDCK^l^	CYP3A4 inhibitor	CYP2D6 inhibitor	Lipiniski^m^	Veber^n^	Muegge^o^	PAINS^p^
**2a**	2.95	437.89	4	1	3	54.34	2.83	96.83	88.01	0.999	33.25	110.95	Yes	Yes	Yes	Yes	Yes	0 alerts
**2b**	1.87	508.98	5	1	4	125.46	0.08	98.09	100	0.017	19.77	3.730	Yes	Yes	Yes (1 violation)	Yes	Yes	0

a Note. Log P: logarithm of compound partition coefficient between *n*-octanol and water.

b M.Wt, molecular weight.

c HBA, number of hydrogen bond acceptors.

d HBD, number of hydrogen bond donors.

e NROTB, number of rotatable bonds.

f TPSA, polar surface area. Drug-like TPSA <140–150 A^2^.

g S, aqueous solubility (mg/L).

h HIA, human intestinal absorption. HIA values <20% (poorly absorbed), values ≈20–70% (moderately absorbed), and values >70% (well absorbed) (Yee 2011).

i PPB, plasma protein binding. PPB values <90% (poorly bound) and values >90% (strongly bound) (https://preadmet.bmdrc.kr/adme/ (accessed August 14, 2020).

j BBB, blood–brain barrier penetration. BBB values <0.1 (low CNS absorption), values ≈0.1–2 (medium CNS absorption) and values >2 (high CNS absorption (Ma et al., 2005).

k Caco2, permeability through cells derived from human colon adenocarcinoma. PCaco2 values <4 nm/s (low permeability), values ≈4–70 nm/s (medium permeability) and values >70 nm/s (high permeability) (Yamashita et al., 2000; Irvine et al., 1999; and; Yazdanian et al., 1998).

l MDCK, permeability through Madin–Darby canine kidney cells. PMDCK values <25 nm/s (low permeability), values ≈25–500 nm/s (medium permeability), and values >500 nm/s (high permeability) (Irvine et al., 1999).

m Lipinski rule: log P ≤ 5, M. Wt ≤ 500 Da, HBA ≤ 10 and HBD ≤ 5 (Lipinski et al., 2017).

n Veber rule: NROTB ≤ 10 and TPSA ≤ 140 (Veber et al., 2002).

o Muegge rule: -2 ≤log P ≤ 5, 200 ≤ M. Wt ≤ 600 Da, TPSA ≤ 150, Num. rings ≤ 7, Num. carbons > 4, Num. heteroatom > 1, NROTB ≤ 15, HBA ≤ 10 and HBD ≤ 5 (Muegge et al., 2011).

## Conclusion

The current study portrays the design, synthesis, characterization, mechanistic study of 32CA reaction and biological evaluation of novel spirooxindole derivatives as dual MDM2 and Bcl2 inhibitors. The supramolecular structure of the studied compound is analyzed using Hirshfeld calculations. The calculated molecular geometry of the studied compound agrees well with the experimental x-ray structure. Also, calculated NMR spectra are in good agreement with the experimental data. Different electronic and reactivity descriptors were calculated and discussed. MTT assay revealed promising anticancer potencies, especially **2b** that was superior to 5-FU against the screened cells with its safe dose. Compound **2b** induced apoptosis-dependent anticancer activity up to 43.08% (superior to 5-FU), activated p53 by up to 47%, downregulated Bcl2 gene by 1.25 fold, and upregulated p21 by two folds in the treated cancer cells. Docking simulations of the synthesized compounds within MDM2 predicted common key interactions between the synthesized derivatives and the reference inhibitor. In light of the aforementioned data, we hope that further research in this area may allow introduction of the next-generation clinical-stage p53-MDM2 inhibitors in the near future.

## Experimental

### Materials and equipment

^1^H-NMR and ^13^C-NMR spectra of spiroxindole analogs **2a, b** were recorded on CDCl_3_ using a JEOL 400 MHz spectrometer (JEOL Ltd., Tokyo, Japan) at room temperature. Mass spectra were recorded on JMS-600 H JEOL spectrometer (JEOL Ltd., Tokyo, Japan). X-Ray crystallographic analysis was collected by using a Bruker SMART APEX II D8 Venture diffractometer at Karachi University. Note for refractive index (specific rotation) measurement: The samples were prepared in 10 ml, then the concentration was calculated in g/100 ml, and a 100-mm polarimeter tube was used. The instrument used was A.KRÜSS Optronic P8000-PT digital polarimeter.

(*E*)-3-(3-Fluorophenyl)-1-(1-methyl-1*H*-pyrrol-2-yl)prop-2-en-1-one **1a** and (*E*)-1-(1-methyl-1*H*-pyrrol-2-yl)-3-(3-nitrophenyl)prop-2-en-1-one **1b** were synthesized according to the literature, and the spectrum is in good agreement with the reported (Yenupuri, et al., 2014).

#### Synthesis of the spiroxindole derivatives **2a, b**

A mixture of *N*-methyl pyrrole-based chalcone **1a, b** (0.5 mmol), 5-Clisatin (0.5 mmol), and the appropriate secondary amine sarcosine/thioproline (0.5 mmol) in methanol (10 ml) was refluxed for 3 h. After completion of the reaction as evident from tlc, the final product was precipitated, and the resulting solid was filtered and recrystallized from DCM/ethanol to afford a pure product.

(3*S*,3′*R*,4′*S*)-5-Chloro-4′-(3-fluorophenyl)-1′-methyl-3′-(1-methyl-1*H*-pyrrole-2-carbonyl)spiro [indoline-3,2′-pyrrolidin]-2-one **2a**

R_f_ = 0.5; eluent: (50% ethyl acetate: hexane); m. p.: 203°C; ^1^H-NMR (CDCl_3_, 400 MHz): *δ* 7.83 (S, NH, 1H), 7.25–7.18 (m, aromatic-H, 4 H), 7.04 (dd, *J* = 8.08, 2.16 Hz, pyrrole-H), 6.91 (m, Ph-H, 1H), 6.60 (s, Ph-H, 1H), 6.54 (d, *J* = 8.28 Hz, pyrrole-H, 2H), 5.83 (t, *J* = 2.08 Hz, Pyrrole-H, 1H), 4.46–4.39 (m, pyrrolidine-H, 1H), 4.18 (d, *J* = 9.56 Hz, pyrrolidine-H, 1H), 3.60–3.55 (t overlaid s, pyrrolidine-H, and methyl, 4H), 3.44–3.40 (t, *J* = 7.28 Hz, pyrrolidine-H, 1H), 2.23 (s, methyl, 3H); ^13^C-NMR (CDCl_3_, 400 MHz): *δ* 186.0 (CO), 179.8(CO), 164.3, 161.8, 143.8, 139.2, 131.7, 129.6, 129.0, 127.9, 127.0, 123.9, 118.8, 115.0, 113.9, 11.7, 110.1, 107.9, 74.2, 63.1, 60.2, 43.7, 37.1, 35.1; HRMS (EI) calcd for C_24_H_21_ClFN_3_O_2_ (M^+^): 437.1300. Found: 437.1311; $\;{\left[\alpha \right]}_{D}^{25}$ = –2.26° (c 0.053, MeOH); UV-Vis (λmax; EtOH): 209, 257, and 293 nm.

(3*S*,6′*R*,7′*S*)-5-Chloro-6′-(1-methyl-1*H*-pyrrole-2-carbonyl)-7′-(3-nitrophenyl)-1′,6′,7′,7a′-tetrahydro-3′*H*-spiro [indoline-3,5′-pyrrolo [1,2-*c*]thiazol]-2-one **2b**

R_f_ = 0.25; eluent: (50% ethyl acetate: hexane); m.p.: 130°C; ^1^H-NMR (CDCl_3_, 400 MHz): *δ* 8.52 (S, NH, 1H), 8.50 (s, Ph-H, 1H), 8.11 (d, *J* = 8.00 Hz, Ph-H, 1H), 7.86 (d, *J* = 8.00 Hz, Ph-H, 1H), 7.66 (d, 2.02 Hz, Pyrrole-H, 1H), 7.51 (t, *J* = 8.04 Hz, Ph-H, 1H), 7.19 (dd, *J* = 8.04, 2.02 Hz, pyrrole-H, 1H), 6.79 (dd, *J* = 4.36, 1.36 Hz, Ph-H, 1H), 6.68 (dd, *J* = 8.02, 1.88 Hz, 2.16 Hz, Ph-H, 1H), 6.56 (t, *J* = 2.02 Hz, Ph-H, 1H), 5.88 (t, *J* = 3.36 Hz, pyrrole-H, 1H), 4.46 (d, *J* = 11.64 Hz, pyrrolidine-H, 1H), 4.36–4.33 (m, pyrrolidine-H, 1H), 4.05 (d, *J* = 11.76 Hz, pyrrolidine-H, 1H), 3.90 (d, *J* = 10.32 Hz, pyrrolidine-H, 1H), 3.53 (d, *J* = 10.52 Hz, pyrrolidine-H, 1H), 3.53 (s, thiazolidine-CH_2_, 2H), 3.33 (s, methyl, 3H), 3.09 (dd, *J* = 11.72, 5.96 Hz, thiazolidine-H, 1H), 3.00 (dd, *J* = 11.76, 2.60 Hz, pyrrolidine-H, 1H), ^13^C-NMR (CDCl_3_, 400 MHz): *δ* 183.7, 180.1, 148.7, 141.3, 139.2, 135.0, 132.1, 130.4, 130.0, 129.0, 128.9, 127.8, 125.2, 123.2, 122.6, 120.0, 110.8, 108.2, 75.1, 74.4, 63.2, 54.5, 50.9, 50.3, 36.8, 36.4; (HRMS (EI) calcd for C_25_H_21_ClN_4_O_4_S (M^+^): 508.1018. Found: 508.1075; $\;{\left[\alpha \right]}_{D}^{25}$ = +7.99° (c 0.044, MeOH); UV-Vis (λmax; EtOH): 209, 256, and 293 nm.

### Single-crystal x-ray diffraction analysis

Single-crystal x-Ray diffraction analysis has been provided in supplementary information.

### Computational methods and Hirshfeld surface analysis

Hirshfeld surface analysis and computational methods have been provided in the supplementary information.

### Biological evaluation assays

Biological evaluation assays including cytotoxicity evaluation, anticancer evaluation, flow cytometric analysis of apoptosis, immunohistochemical detection of tumor suppressor protein (p53), qRT-PCR analysis of p21 and Bcl2 gene expression, and statistical analysis, have been provided in the Supplementary Information.

### Molecular docking protocol

Molecular docking protocol has been provided in the Supplementary Information.

## Data Availability

The datasets presented in this study can be found in online repositories. The names of the repository/repositories and accession number(s) can be found below: The Cambridge Crystallographic Data Centre (CCDC); CCDC numbers: 2012711 and 2012712.
