# Supplementary material for: Design, Synthesis, Chemical and Biochemical Insights Into Novel Hybrid Spirooxindole-Based p53-MDM2 Inhibitors With Potential Bcl2 Signaling Attenuation
Source: Front Chem. 2021 Dec 14;9:735236. doi: 10.3389/fchem.2021.735236 (PMC8713455; doi:10.3389/fchem.2021.735236)
Supplement: Supplementary file 1 [file DataSheet1.docx]

**Design, Synthesis, Chemical and Biochemical Insights into Novel Hybrid Spirooxindoles-Based p53-MDM2 Inhibitors with Potential Bcl2 Signaling Attenuation**

***Yasmine M. Abdel Aziz, ^1^ Gehad Lotfy, ^1^ Mohamed M. Said, ^1^ El Sayed H. El Ashry,^2^ El Sayed H. El Tamany,^3^ Saied M. Soliman, ^2^ Marwa M. Abu-Serie,^4^ Mohamed Teleb,^5^ Sammer Yousuf,^6^ Alexander Dömling,^7^ Luis R. Domingo,^8^* *and Assem Barakat^9^****

^1^Pharmaceutical Organic Chemistry Department, Faculty of Pharmacy, Suez Canal University, Ismailia, Egypt.

^2^Department of Chemistry, Faculty of Science, Alexandria University, Ibrahimia, Alexandria, Egypt.

^3^Department of Chemistry, Faculty of Science, Suez Canal University, Ismailia, Egypt.

^4^Medical Biotechnology Department, Genetic Engineering and Biotechnology Research Institute, City of Scientific Research and Technological Applications (SRTA-City), Egypt.

^5^Department of Pharmaceutical Chemistry, Faculty of Pharmacy, Alexandria University, Alexandria, Egypt.

^6^H.E.J. Research Institute of Chemistry, International Center for Chemical and Biological Sciences, University of Karachi, Karachi, Pakistan.

^7^Department of Drug Design, Groningen Research Institute of Pharmacy, University of Groningen, Groningen, The Netherlands.

^8^Department of Organic Chemistry, University of Valencia, Valencia, Spain.

^9^ Department of Chemistry, College of Science, King Saud University, Riyadh, Saudi Arabia.

Corresponding Authors

***** Author to whom correspondence should be addressed; E-Mail: [ambarakat@ksu.edu.sa](mailto:ambarakat@ksu.edu.sa). Tel.: +966-11467-5901 (A.B.); Fax: +966-11467-5992 (A.B.).

**Table of contents:**

1. Single-Crystal X-Ray Diffraction Analysis
2. Computational Methods
3. Hirshfeld surface analysis
4. Biological evaluation

- Cytotoxicity evaluation
- Anticancer evaluation
- Flow cytometric analysis of apoptosis
- Immunohistochemical detection of tumor suppressor protein (p53)
- qRT-PCR analysis of p21 and Bcl2 gene expression
- Statistical analysis

1. Molecular modeling studies
2. Reference.
3. Copies of Spectrum.
4. Computational studies assistant figures.

**Single-Crystal X-Ray Diffraction Analysis**

X-Ray diffraction data of target molecule was collected on Bruker D8 Venture Diffrectometer with the aid of Cu Kα (λ = [0.71073](file:///C:\Users\Dr.Sammar\Downloads\ab517%20_diffrn_radiation_wavelength)Å) radiation source and CMOS Photon 100 detector 100 (Spek, L.A. 2002). Data integration, and reduction were carried out by APEX 3 software package (Bruker, A. 1998) followed by use of SHELXL programs to solve and refine the final structure [2] (Table 1). Geometrical calculations and inter-molecular interaction studies were performed on *PLATON* and *Mercury* 3.10.1, respectively (Sheldrick, G.M. 2015; and Macrae, C. F., et al., 2008).

**Table S1**: Crystal and experimental data of **2a,b**

| **Crystal data** | **2a** | **2b** |
| --- | --- | --- |
| **Fwt** | [C_48_H_42_Cl_2_F_2_N_6_O_4_](file:///M:\5law\Downloads\a%20_chemical_formula_moiety) | [C_25_H_21_ClN_4_O_4_S·CH_4_O](file:///M:\5law\Downloads\a%20_chemical_formula_moiety) |
| **Mwt** | [875.78](file:///M:\5law\Downloads\a%20_chemical_formula_weight) | [541.02](file:///M:\5law\Downloads\a%20_chemical_formula_weight) |
| **T** | [100](file:///M:\5law\Downloads\a%20_cell_measurement_temperature) | [100](file:///C:\assem\MSc%20students\GEAHD\PhD\N-Methylpyrrolidine%20project\Xray\a%20_cell_measurement_temperature) |
| **λ (**[**Mo *K*α**](file:///C:\Users\pc754\AppData\Users\7OBY\AppData\pc754\Documents\a%20_diffrn_radiation_type) **radiation)** | λ = [0.71073](file:///C:\Users\Dr.Sammar\Downloads\ab517%20_diffrn_radiation_wavelength) Å | λ = [0.71073](file:///C:\Users\Dr.Sammar\Downloads\ab517%20_diffrn_radiation_wavelength) Å |
| **Crystal system/Space group** | [Triclinic](file:///M:\5law\Downloads\a%20_symmetry_cell_setting), [*P*](file:///M:\5law\Downloads\a%20_symmetry_space_group_name_H-M) | [Monoclinic](file:///M:\5law\Downloads\a%20_symmetry_cell_setting), [*P*2_1_/*c*](file:///M:\5law\Downloads\a%20_symmetry_space_group_name_H-M) |
| **Unit cell dimensions** | ***a*** = [11.3633 (5)](file:///C:\assem\MSc%20students\GEAHD\PhD\N-Methylpyrrolidine%20project\Xray\a%20_cell_length_a) Å  ***b*** = [13.7483 (6)](file:///C:\assem\MSc%20students\GEAHD\PhD\N-Methylpyrrolidine%20project\Xray\a%20_cell_length_b) Å  ***c*** = [15.5909 (6)](file:///C:\assem\MSc%20students\GEAHD\PhD\N-Methylpyrrolidine%20project\Xray\a%20_cell_length_c) Å  α = [101.941 (2)](file:///C:\assem\MSc%20students\GEAHD\PhD\N-Methylpyrrolidine%20project\Xray\a%20_cell_angle_alpha)  β = [108.417 (2)](file:///C:\assem\MSc%20students\GEAHD\PhD\N-Methylpyrrolidine%20project\Xray\a%20_cell_angle_beta)  γ = [108.367 (2)](file:///C:\assem\MSc%20students\GEAHD\PhD\N-Methylpyrrolidine%20project\Xray\a%20_cell_angle_gamma) | ***a*** = [8.0614 (5)](file:///M:\5law\Downloads\a%20_cell_length_a) Å  ***b*** = [10.7988 (7)](file:///M:\5law\Downloads\a%20_cell_length_b) Å  ***c*** = [29.0170 (19)](file:///M:\5law\Downloads\a%20_cell_length_c) Å  β = [92.544 (3)](file:///M:\5law\Downloads\a%20_cell_angle_beta)° |
| **Volume** | [2062.30 (16)](file:///M:\5law\Downloads\a%20_cell_volume) Å^3^ | [2523.5 (3)](file:///M:\5law\Downloads\a%20_cell_volume) Å^3^ |
| **Z** | 2 | 4 |
| **Density (calculated)** | [1.410](file:///M:\5law\Downloads\a%20_exptl_crystal_density_diffrn) Mg m^−3^ | [1.424](file:///M:\5law\Downloads\a%20_exptl_crystal_density_diffrn) Mg m^−3^ |
| **Absorption coefficient** | [1.95](file:///M:\5law\Downloads\a%20_exptl_absorpt_coefficient_mu) mm^−1^ | [2.50](file:///M:\5law\Downloads\a%20_exptl_absorpt_coefficient_mu) mm^−1^ |
| **Crystal size** | [0.13](file:///M:\5law\Downloads\a%20_exptl_crystal_size_max) × [0.10](file:///M:\5law\Downloads\a%20_exptl_crystal_size_mid) × [0.03](file:///M:\5law\Downloads\a%20_exptl_crystal_size_min)mm | [0.08](file:///M:\5law\Downloads\a%20_exptl_crystal_size_max) × [0.05](file:///M:\5law\Downloads\a%20_exptl_crystal_size_mid) × [0.03](file:///M:\5law\Downloads\a%20_exptl_crystal_size_min) mm |
| **Theta range for data collection** | θ_max_ =[68.4](file:///C:\assem\MSc%20students\GEAHD\PhD\N-Methylpyrrolidine%20project\Xray\a%20_cell_measurement_theta_max)° , θ_min_ = [3.2](file:///M:\5law\Downloads\a%20_cell_measurement_theta_min) | θ_max_ = [68.2](file:///M:\5law\Downloads\a%20_diffrn_reflns_theta_max)°, θ_min_ = [3.1](file:///M:\5law\Downloads\a%20_diffrn_reflns_theta_min)° |
| **Goodness-of-fit on *F^2^*** | [1.02](file:///C:\assem\MSc%20students\GEAHD\PhD\N-Methylpyrrolidine%20project\Xray\a%20_refine_ls_goodness_of_fit_ref) mm^−1^ | [1.07](file:///M:\5law\Downloads\a%20_refine_ls_goodness_of_fit_ref)mm^−1^ |
| **Diffractometer** | [Bruker APEX-II D8 Venture  diffractometer](file:///C:\Users\User\AppData\Local\Temp\a_a%20_diffrn_measurement_device_type) | [Bruker APEX-II D8 Venture  diffractometer](file:///C:\Users\User\AppData\Local\Temp\a_a%20_diffrn_measurement_device_type) |
| **Refinement method** | Refinement on [F2](file:///C:\Users\ambarakat\AppData\Roaming\Microsoft\Word\a_a%20_refine_ls_structure_factor_coef) / [H atoms treated by a mixture of independent and constrained refinement](file:///C:\Users\User\AppData\Local\Temp\ab440%20_refine_ls_hydrogen_treatment) | Refinement on [F2](file:///C:\Users\ambarakat\AppData\Roaming\Microsoft\Word\a_a%20_refine_ls_structure_factor_coef) / [H atoms treated by a mixture of independent and constrained refinement](file:///C:\Users\User\AppData\Local\Temp\ab440%20_refine_ls_hydrogen_treatment) |
| **Data/restraints/ parameters** | [7558](file:///M:\5law\Downloads\a%20_refine_ls_number_reflns)/0/576 | [4615](file:///M:\5law\Downloads\a%20_refine_ls_number_reflns)/0/340 |
| ***R*_int_** | [0.037](file:///M:\5law\Downloads\a%20_diffrn_reflns_av_R_equivalents) | [0.073](file:///M:\5law\Downloads\a%20_diffrn_reflns_av_R_equivalents) |
| ***R*[*F*^2^ > 2σ(*F*^2^)]** | [0.042](file:///M:\5law\Downloads\a%20_refine_ls_R_factor_gt) | [0.043](file:///M:\5law\Downloads\a%20_refine_ls_R_factor_gt) |
| ***wR*(*F*^2^)** | [0.119](file:///C:\assem\MSc%20students\GEAHD\PhD\N-Methylpyrrolidine%20project\Xray\a%20_refine_ls_wR_factor_ref) | [1.07](file:///M:\5law\Downloads\a%20_refine_ls_goodness_of_fit_ref) |
| **Δρ_max_/Δρ_min_** | [0.66](file:///M:\5law\Downloads\a%20_refine_diff_density_max) e Å^−3^/ [−0.55](file:///M:\5law\Downloads\a%20_refine_diff_density_min) e Å^−3^ | [0.74](file:///M:\5law\Downloads\a%20_refine_diff_density_max) e Å^−3^/ [−0.47](file:///M:\5law\Downloads\a%20_refine_diff_density_min) e Å^−3^ |
| **CCDC Number** | 2012711 | 2012712 |

**Table S2**: The list of selected hydrogen bonds geometry Å in **2a** is given below:

| **D** | **H** | **A** | **D-H** | **H…A** | **D…A** | **D-H…A** |
| --- | --- | --- | --- | --- | --- | --- |
| N2 | H2A | O3 | 0.88 | 2.04 | 2.9046 | 168 |
| N3 | H3A | N1 | 0.89 | 2.20 | 3.0784 | 169 |
| C14 | H3 | O4 | 0.99 | 2.56 | 3.2510 | 126 |
| C9 | H12 | O4 | 0.95 | 2.57 | 3.3412 | 139 |
| C14 | H15 | O4 | 0.99 | 2.59 | 3.4922 | 152 |
| C40 | H24 | F2 | 0.98 | 2.45 | 3.4083 | 166 |
| C28 | H38 | N4 | 0.95 | 2.53 | 3.4292 | 158 |

Symmetric codes: −*x*, −*y*+1, −*z*+1; −*x*+1, −*y*+2, −*z*+1; *x*−1, *y*, *z*; −*x*+2, −*y*+2, −*z*+2

**Table S3:** The list of selected hydrogen bonds geometry Å in **2b** is given below:

| **D** | **H** | **A** | **D-H** | **H…A** | **D…A** | **D-H…A** |
| --- | --- | --- | --- | --- | --- | --- |
| N4 | H4A | O5 | 0.88 | 1.94 | 2.8199 | 173 |
| C11 | H1 | O5 | 0.95 | 2.58 | 3.4252 | 148 |
| C20 | H11 | O2 | 0.98 | 2.34 | 3.0474 | 128 |
| C26 | H19 | O4 | 0.95 | 2.42 | 3.2463 | 145 |

Symmetric codes: −*x*+1, *y*+1/2, −z+3/2; *x*, *y*+1, z.

**Computational Methods**

The *ω*B97X-D (Chai, J.-D., et al., functional, together with the standard 6-311G(d,p) (Hehre, M.J., et al., 1986) basis set, were used throughout the MEDT study. Solvent effects of methanol were taken into account by full optimization of the gas phase structures at the same computational level using the polarizable continuum model (PCM) (Tomasi, J., et al., 1994; Simkin, B.Ya., et al., 1995) in the framework of the self-consistent reaction field (SCRF) (Cossi, M., et al., 1996; Cances, E., et al., 1997; and Barone, V., et al., 1998). The GEDT (Domingo, L.R. 2014). values were computed by using the equation GEDT(f) = Σq_f_, were q are the natural charges (Reed, A.E., et al., 1985 & 1988) of the atoms belonging to one of the two frameworks (f) at the TS geometries. Global and local CDFT indices (Domingo, L.R. et al., 2016; Parr, R.G., et al.1989) were calculated by using the equations given in reference (Domingo, L.R. et al., 2016). The Gaussian 16 suite of programs was used to perform the calculations. (Frisch, M. J. et al. 2016).

In addition, the DFT calculations on the final products were performed using B3LYP/6-31G(d,p) method. The resulting optimized structures showed no imaginary frequencies. Natural population analysis was performed using NBO 3.1 program (Reed, A.E., et al.,1988).

**Hirshfeld surface analysis**

The topology analyses were performed using Crystal Explorer 17.5 program (Turner, M. J., et al., 2017) in order to determine the percentages of the different intermolecular interactions in the crystal structure of the studied compound.

**Table S4:** The calculated bond distances of **2a**.

| **Bond** | **Calc.** | **Exp.** | **Bond** | **Calc.** | **Exp.** |
| --- | --- | --- | --- | --- | --- |
|  | **2a_F1** |  | **2a_F2** |  |  |
| R(1-32) | 1.227 | 1.218 | R(1-27) | 1.764 | 1.752 |
| R(2-25) | 1.763 | 1.736 | R(2-28) | 1.213 | 1.219 |
| R(3-22) | 1.354 | 1.362 | R(3-36) | 1.226 | 1.223 |
| R(4-52) | 1.208 | 1.227 | R(4-10) | 1.354 | 1.394 |
| R(5-14) | 1.469 | 1.472 | R(5-20) | 1.402 | 1.406 |
| R(5-29) | 1.458 | 1.481 | R(5-28) | 1.377 | 1.364 |
| R(5-48) | 1.454 | 1.457 | R(6-18) | 1.465 | 1.467 |
| R(6-12) | 1.401 | 1.407 | R(6-29) | 1.455 | 1.456 |
| R(6-52) | 1.376 | 1.349 | R(6-33) | 1.458 | 1.46 |
| R(8-10) | 1.397 | 1.391 | R(7-37) | 1.392 | 1.389 |
| R(8-25) | 1.389 | 1.383 | R(7-42) | 1.358 | 1.354 |
| R(10-12) | 1.386 | 1.383 | R(7-44) | 1.462 | 1.458 |
| R(12-13) | 1.403 | 1.397 | R(8-10) | 1.385 | 1.375 |
| R(13-14) | 1.529 | 1.522 | R(8-48) | 1.394 | 1.385 |
| R(13-26) | 1.387 | 1.381 | R(10-11) | 1.387 | 1.383 |
| R(14-15) | 1.568 | 1.557 | R(11-13) | 1.397 | 1.397 |
| R(14-52) | 1.563 | 1.553 | R(13-14) | 1.514 | 1.508 |
| R(15-17) | 1.562 | 1.547 | R(13-50) | 1.402 | 1.393 |
| R(15-32) | 1.535 | 1.556 | R(14-16) | 1.554 | 1.547 |
| R(17-19) | 1.517 | 1.513 | R(14-33) | 1.534 | 1.528 |
| R(17-29) | 1.544 | 1.542 | R(16-18) | 1.584 | 1.578 |
| R(19-20) | 1.402 | 1.392 | R(16-36) | 1.533 | 1.531 |
| R(19-46) | 1.398 | 1.398 | R(18-19) | 1.518 | 1.512 |
| R(20-22) | 1.384 | 1.379 | R(18-28) | 1.569 | 1.557 |
| R(22-23) | 1.388 | 1.372 | R(19-20) | 1.4 | 1.399 |
| R(23-44) | 1.391 | 1.383 | R(19-25) | 1.384 | 1.381 |
| R(25-26) | 1.398 | 1.396 | R(20-21) | 1.387 | 1.381 |
| R(28-33) | 1.393 | 1.41 | R(21-23) | 1.398 | 1.389 |
| R(28-38) | 1.358 | 1.341 | R(23-27) | 1.39 | 1.384 |
| R(28-40) | 1.462 | 1.451 | R(25-27) | 1.397 | 1.392 |
| R(32-33) | 1.454 | 1.444 | R(36-37) | 1.454 | 1.455 |
| R(33-34) | 1.399 | 1.374 | R(37-38) | 1.399 | 1.391 |
| R(34-36) | 1.403 | 1.424 | R(38-40) | 1.403 | 1.403 |
| R(36-38) | 1.386 | 1.373 | R(40-42) | 1.386 | 1.366 |
| R(44-46) | 1.395 | 1.389 | R(48-50) | 1.392 | 1.383 |

**Table S5:** The calculated bond distances of **2b**.

| **Bond** | **Calc.** | **Exp.** | **Bond** | **Calc.** | **Exp.** |
| --- | --- | --- | --- | --- | --- |
| **2b_MeOH** | | | **2b_noMeOH** | | |
| R(1-24) | 1.76 | 1.749 | R(1-24) | 1.761 | 1.749 |
| R(2-45) | 1.866 | 1.83 | R(2-45) | 1.866 | 1.83 |
| R(2-48) | 1.836 | 1.804 | R(2-48) | 1.834 | 1.804 |
| R(3-11) | 1.224 | 1.242 | R(3-11) | 1.224 | 1.242 |
| R(4-33) | 1.226 | 1.229 | R(4-33) | 1.227 | 1.229 |
| R(5-53) | 1.216 | 1.223 | R(5-53) | 1.208 | 1.223 |
| R(6-11) | 1.224 | 1.224 | R(6-11) | 1.223 | 1.224 |
| R(7-34) | 1.393 | 1.384 | R(7-34) | 1.393 | 1.384 |
| R(7-35) | 1.358 | 1.352 | R(7-35) | 1.357 | 1.352 |
| R(7-41) | 1.462 | 1.452 | R(7-41) | 1.462 | 1.452 |
| R(8-20) | 1.467 | 1.48 | R(8-20) | 1.473 | 1.48 |
| R(8-45) | 1.439 | 1.454 | R(8-45) | 1.44 | 1.454 |
| R(8-51) | 1.48 | 1.485 | R(8-51) | 1.474 | 1.485 |
| R(9-53) | 1.366 | 1.346 | R(9-53) | 1.376 | 1.346 |
| R(9-54) | 1.404 | 1.408 | R(9-54) | 1.4 | 1.408 |
| R(11-12) | 1.482 | 1.467 | R(11-12) | 1.482 | 1.467 |
| R(12-13) | 1.39 | 1.382 | R(12-13) | 1.389 | 1.382 |
| R(12-31) | 1.391 | 1.38 | R(12-31) | 1.39 | 1.38 |
| R(13-15) | 1.4 | 1.389 | R(13-15) | 1.398 | 1.389 |
| R(15-16) | 1.514 | 1.509 | R(15-16) | 1.513 | 1.509 |
| R(15-27) | 1.4 | 1.395 | R(15-27) | 1.4 | 1.395 |
| R(16-18) | 1.542 | 1.532 | R(16-18) | 1.539 | 1.532 |
| R(16-51) | 1.539 | 1.536 | R(16-51) | 1.54 | 1.536 |
| R(18-20) | 1.574 | 1.565 | R(18-20) | 1.57 | 1.565 |
| R(18-33) | 1.533 | 1.524 | R(18-33) | 1.535 | 1.524 |
| R(20-21) | 1.528 | 1.523 | R(20-21) | 1.525 | 1.523 |
| R(20-53) | 1.566 | 1.555 | R(20-53) | 1.569 | 1.555 |
| R(21-22) | 1.386 | 1.385 | R(21-22) | 1.387 | 1.385 |
| R(21-54) | 1.401 | 1.4 | R(21-54) | 1.403 | 1.4 |
| R(22-24) | 1.397 | 1.389 | R(22-24) | 1.397 | 1.389 |
| R(24-25) | 1.39 | 1.39 | R(24-25) | 1.39 | 1.39 |
| R(25-55) | 1.397 | 1.39 | R(25-55) | 1.397 | 1.39 |
| R(27-29) | 1.394 | 1.391 | R(27-29) | 1.394 | 1.391 |
| R(29-31) | 1.389 | 1.385 | R(29-31) | 1.39 | 1.385 |
| R(33-34) | 1.453 | 1.459 | R(33-34) | 1.451 | 1.459 |
| R(34-39) | 1.4 | 1.386 | R(34-39) | 1.4 | 1.386 |
| R(35-37) | 1.387 | 1.366 | R(35-37) | 1.387 | 1.366 |
| R(37-39) | 1.402 | 1.405 | R(37-39) | 1.402 | 1.405 |
| R(48-51) | 1.546 | 1.535 | R(48-51) | 1.547 | 1.535 |
| R(54-55) | 1.386 | 1.377 | R(54-55) | 1.387 | 1.377 |
| R(57-58) | 1.423 | 1.401 |  |  |  |

**Biological evaluation**

***Cytotoxicity evaluation***

Normal human lung fibroblast Wi-38 cell line was used to detect cytotoxicity of the studied compounds. Wi-38 cell line was cultured in DMEM medium-contained 10% fetal bovine serum (FBS), seeded as 5x10^3^ cells per well in 96-well cell culture plate and incubated at 37 ºC in 5% CO_2_ incubator. After 24 h for cell attachment, serial concentrations of the synthetic compounds were incubated with Wi-38 cells for 72 h. Cell viability was assayed by MTT method ([Mosmann, 1983](#_ENREF_122)). Twenty microliters of 5 mg/ml MTT (Sigma, USA) was added to each well and the plate was incubated at 37ºC for 3 h. Then MTT solution was removed, 100 µl DMSO was added and the absorbance of each well was measured with a microplate reader (BMG LabTech, Germany) at 570 nm. The effective safe concentration (EC_100_) value (at 100% cell viability) of the tested compounds was estimated by the Graphpad Instat software.

***Anticancer evaluation***

Anticancer effect of the above-mentioned compounds was assayed using three human cancer cell lines. Triple negative breast cancer cell line (MDA-MB 231) and liver cancer cell line (HepG-2), were cultured in RPMI-1640 (Lonza, USA) supplemented with 10% FBS while colon cancer cell line (Caco-2) was cultured in DMEM (Lonza, USA) contained with 10% FBS. All cancer cells (4x10^3^ cells/well) were seeded in sterile 96-well plates. After 24h, serial concentrations of the tested compounds were incubated with three cancer cell lines for 72 h at 37ºC in 5% CO_2_ incubator. MTT method was done as described above. The half maximal inhibitory concentration (IC_50_) values were calculated using the Graphpad Instat software. Furthermore, cellular morphological changes before and after treatment with the tested compounds were investigated using phase contrast inverted microscope with a digital camera (Olympus, Japan).

***Flow cytometric analysis of apoptosis***

Two investigated compounds were incubated, for 72 h, with MDA-MB231, HepG-2 and Caco-2 cell lines. After trypsinization, the untreated and treated cells were incubated with annexin V/PI for 15 min. Then cells were fixed and incubated with streptavidin-fluorescein (5 µg/mL) for 15 min. The apoptosis-dependent anticancer effect was determined by quantification of annexin-stained apoptotic cells using the FITC signal detector (FL1) against the phycoerythrin emission signal detector (FL2).

***Immunohistochemical detection of tumor suppressor protein (p53)***

After trypsinization, the untreated and treated HepG2 cells were centrifuged and washed with PBS buffer and then 10% formalin in PBS was added to cell pellets. The fixed cell specimens were dehydrated in ascending grades of alcohol and immersed in xylene for one hour (three times) followed by impregnation in melted paraffin to from solid paraffin blocks. Then a rotator microtome was used to cut each block into 3-5 μm thick sections that were transferred into positively charge slides. Slides were dried at 60-70 °C for 1-2 h then dewaxed by immersion in xylene 3 times and rehydrated in descending grading ethanol. After that slides were incubated in 3% H_2_O_2_ for 10 min, washed in PBS buffer twice for 3 min and put in 10 mM citrate buffer (pH) followed by heating for 10-20 min. After cooling and washing in PBS, slides were separately soaked overnight in primary antibody (anti-p53). Slides were washed in PBS, covered with biotinylated goat anti-polyvalent secondary antibody for 10 min and then streptavidin peroxidase was added. After 10 min, substrate of secondary antibody (3,3'-diaminobenzidine) was added followed by washing in PBS and placing in hematoxylin bath for 1-4 min then washing in PBS (1 min) and water (3 min). Percentage of immunostained cells was evaluated by imaging analysis cellSens software of phase contrast microscope (Olympus, Japan).

***qRT-PCR analysis of p21 and Bcl2 gene expression***

Total RNAs of the untreated and two anticancer compounds-treated HepG-2 cells were extracted using Gene JET RNA Purification Kit (Thermo Scientific, USA). The cDNA was synthesized from mRNA using cDNA Synthesis Kit (Thermo Scientific, USA). Real time PCR was performed using SYBR green master mix and specific primers (Forward/Reverse) were 5′-CTGGGGATGTCCGTCAGAAC-3′/5′-GCCATTAGCGCATCACAGT-3′ and 5′-TCCGATCAGGAAGGCTAGAGTT-3′/5′-TCGGTCTCCTAAAAGCAGGC**-**3′ for p21, and Bcl-2 genes, respectively. The 2^−ΔΔCT^ equation was used to estimate the change in gene expressions in the treated cancer cells relative to untreated cancer cells.

***Statistical analysis***

The data are expressed as mean ± standard error of mean (SEM) and the significant values were considered at *p* < 0.05. One-way analysis of variance (ANOVA)-Tukey’s test and T-test were used for evaluating the difference between the mean values of the studied treatments. The analysis was done for three measurements using SPSS software version 16.

**Molecular modeling studies**

Docking simulations were performed employing MOE 2016.0802. The studied compounds were built *in silico* and subjected to energy minimization using the MMFF94x force field at a gradient of 0.01 RMSD. Coordinates for MDM2 was downloaded from RCSB PDB (PDB ID: 5LAW), handled with MOE 2016.0802. Unwanted amino acid chains, solvents, and ligands were eliminated. The preparation procedure was conducted employing “Structure Preparation” module with the default settings. The molecular docking simulations were conducted employing ‘Triangle Matcher’ as the placement method and ‘London dG’ scoring for calculating Gibbs energy for binding.

**Reference**

Barone, V., Cossi, M., Tomasi, J. (1998). Geometry optimization of molecular structures in solution by the polarizable continuum model. *J. Comput. Chem.* *19*, 404-417.

Bruker, A. SAINT Software reference manual. Madison, WI, USA, 1998.

Cances, E., Mennucci, B., Tomasi, J. (1997). A new integral equation formalism for the polarizable continuum model: Theoretical background and applications to isotropic and anisotropic dielectrics. *J. Chem. Phys.*, *107*, 3032-3041.

Chai, J.-D.; Head-Gordon, M. (2008). Long-range corrected hybrid density functionals with damped atom–atom dispersion corrections. *Phys. Chem. Chem. Phys.* *10*, 6615-6620.

Cossi, M., Barone, V., Cammi, R., Tomasi, J. (1996). Ab initio study of solvated molecules: A new implementation of the polarizable continuum model. *Chem. Phys. Lett.*, *255*, 327-335.

Domingo, L.R. (2014). A new C-C bond formation model based on the quantum chemical topology of electron density. *RSC Adv*., *4*, 32415-32428.

Domingo, L.R.; Ríos-Gutiérrez, M.; Pérez, P. (2016). Applications of the conceptual density functional indices to organic chemistry reactivity. *Molecules* *21*, 748.

Frisch, M. J. et all. Gaussian 16, Revision A.03, Gaussian, Inc., Wallingford CT, 2016.

Hehre, M.J., Radom, L., Schleyer, P.v.R., Pople, J. (1986). Ab initio Molecular Orbital Theory, Wiley, New York, 1986.

Macrae, C. F., Bruno, I. J., Chisholm, J. A., Edgington, P. R., McCabe, P., Pidcock, E., Rodriguez-Monge, L., Taylor, R.,van de Streek, J.,Wood, P. A. *J. Appl. Cryst*. **2008**, 41, 466–470.

Mosmann, T. (1983). Use of MTT colorimetric assay to measure cell activation. *J. Immunol. Methods*, *65*(1), p.55.

Parr, R.G., Yang, W. (1989). *Density-Functional Theory of Atoms and Molecules*; Oxford University Press: New York, NY, USA.

Reed, A.E., Weinstock, R.B., Weinhold, F. (1985). Natural population analysis. *J. Chem. Phys.*, *83*, 735-746.

Reed, A.E.; Curtiss, L.A.; Weinhold, F. Intermolecular interactions from a natural bond orbital, donor-acceptor viewpoint. *Chem. Rev.*, **1988**, *88*, 899-926.

Sheldrick, G.M. Crystal structure refinement with SHELXL. *Acta Cryst. C.* **2015**, *71*, 3–8.

Simkin, B.Ya., Sheikhet, I.I. (1995). Quantum chemical and statistical theory of solutions– computational approach, Ellis Horwood: London.

Spek, L.A. Single-crystal structure validation with the program PLATON. J. Appl. Chem. **2002**, *36*, 7–13.

Tomasi, J., Persico, M. (1994). Molecular interactions in solution: and overview of methods based on continuous distributions of the solvent. *Chem. Rev.*, *94*, 2027-2094.

Turner, M. J., McKinnon, J. J., SWolff, K., Grimwood, D. J., Spackman, P. R., Jayatilaka, D., Spackman, M. A. Crystal Explorer17 (2017) University of Western Australia. [http://hirshfeldsurface.net](http://hirshfeldsurface.net/).

NMR and IR spectrum for the compounds **2a** and **2b**


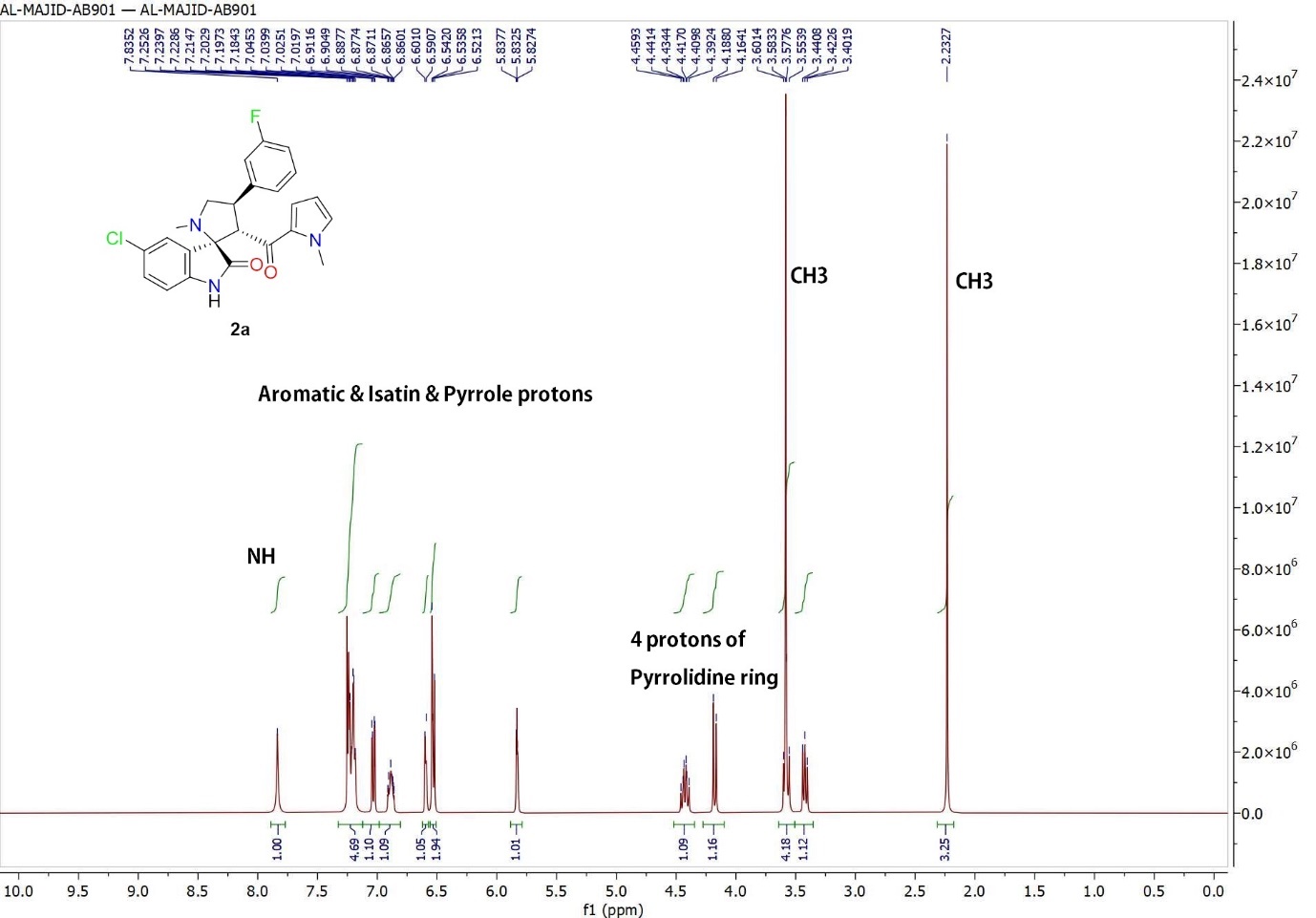


**Figure S1:** ^1^HNMR of **2a**


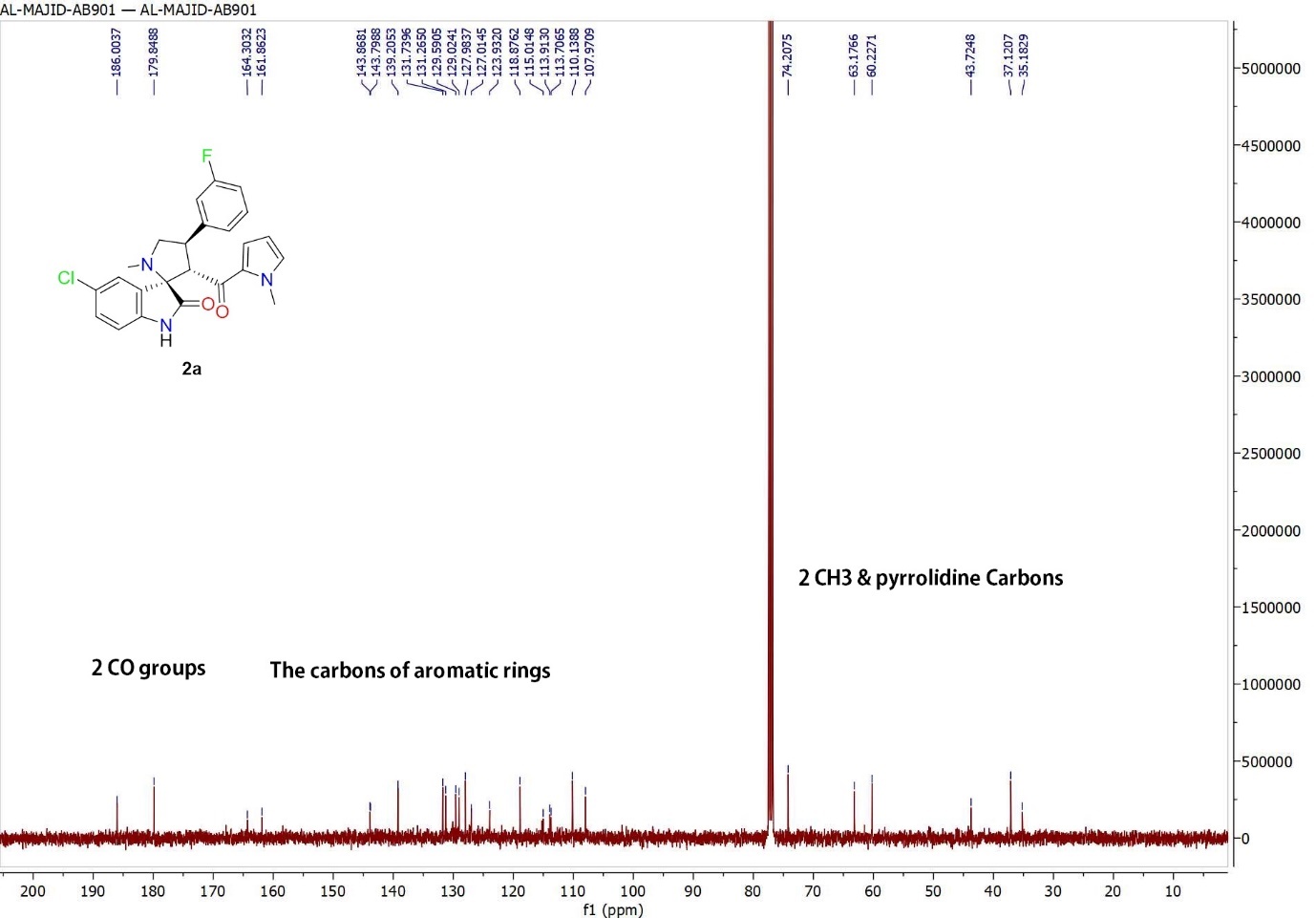


**Figure S2:** ^13^CNMR of **2a**

**
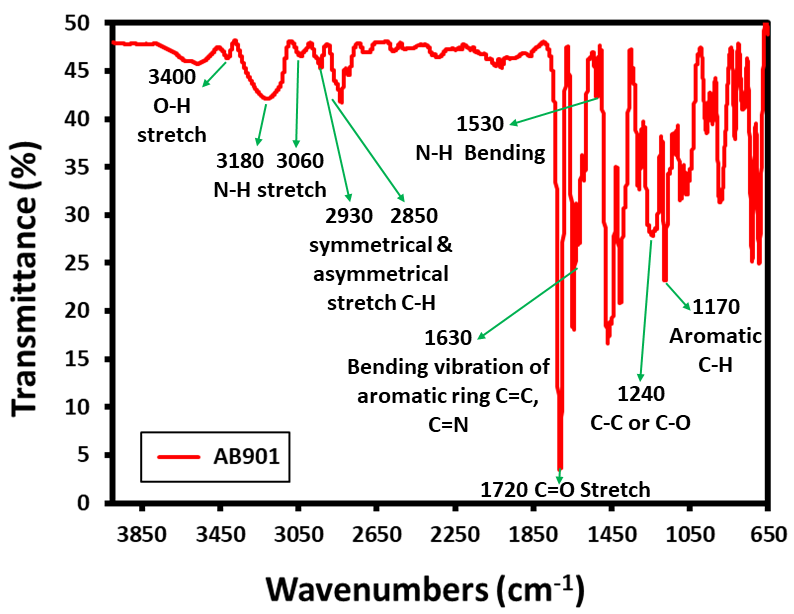
**

**Figure S3:** IR of **2a**


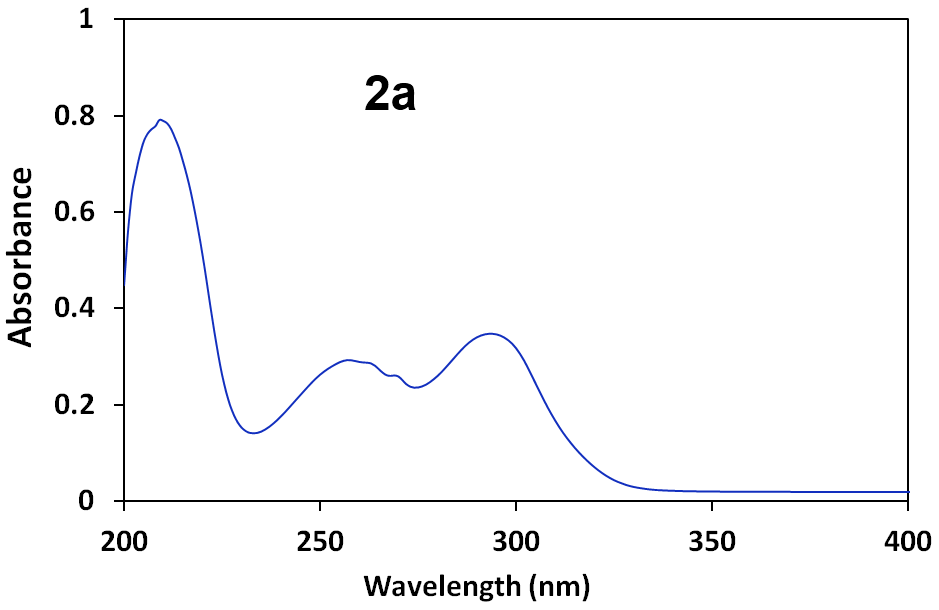


**Figure S4:** UV-Vis of **2a (**in EtOH).


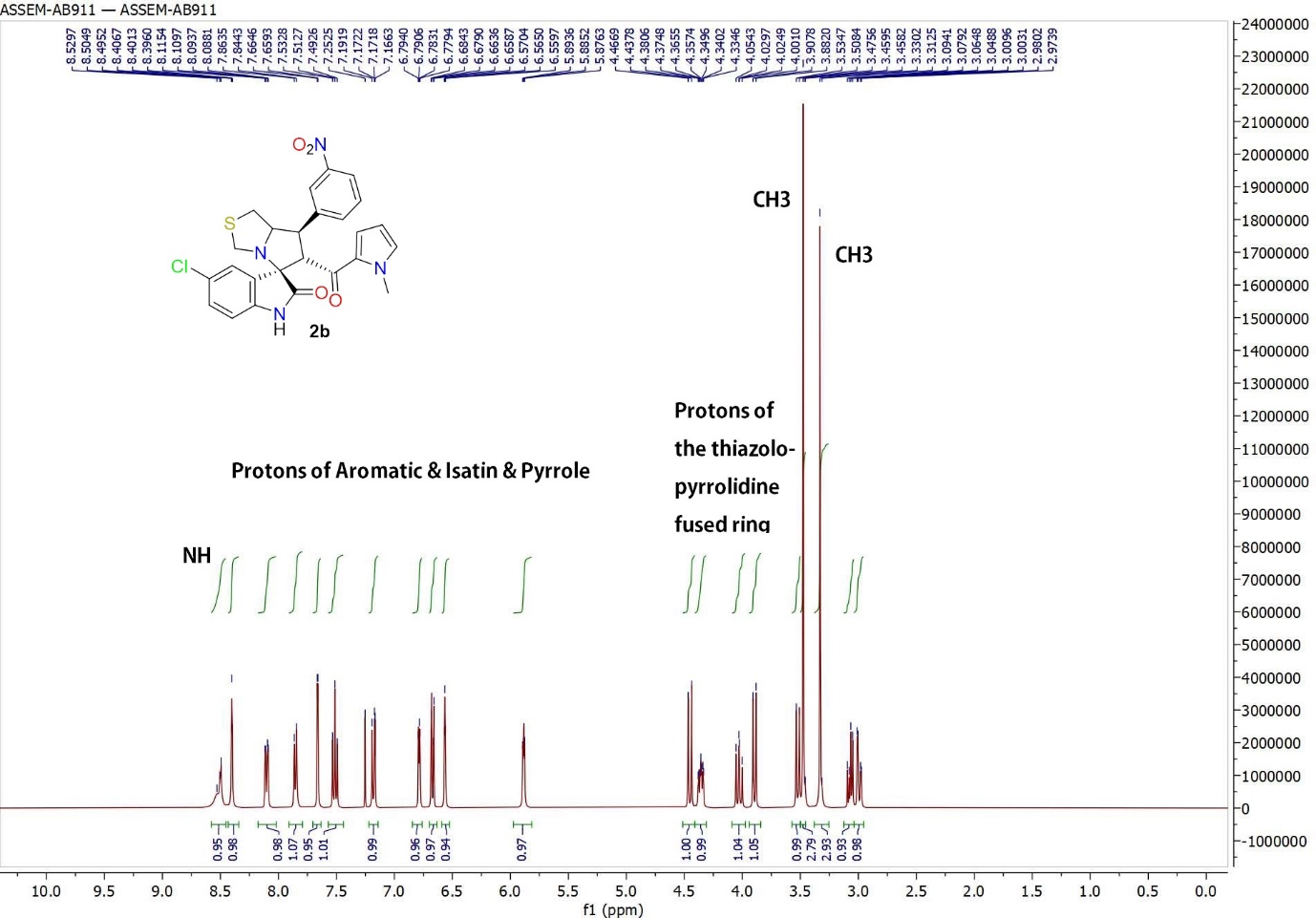


**Figure S5:** ^1^HNMR of **2b**


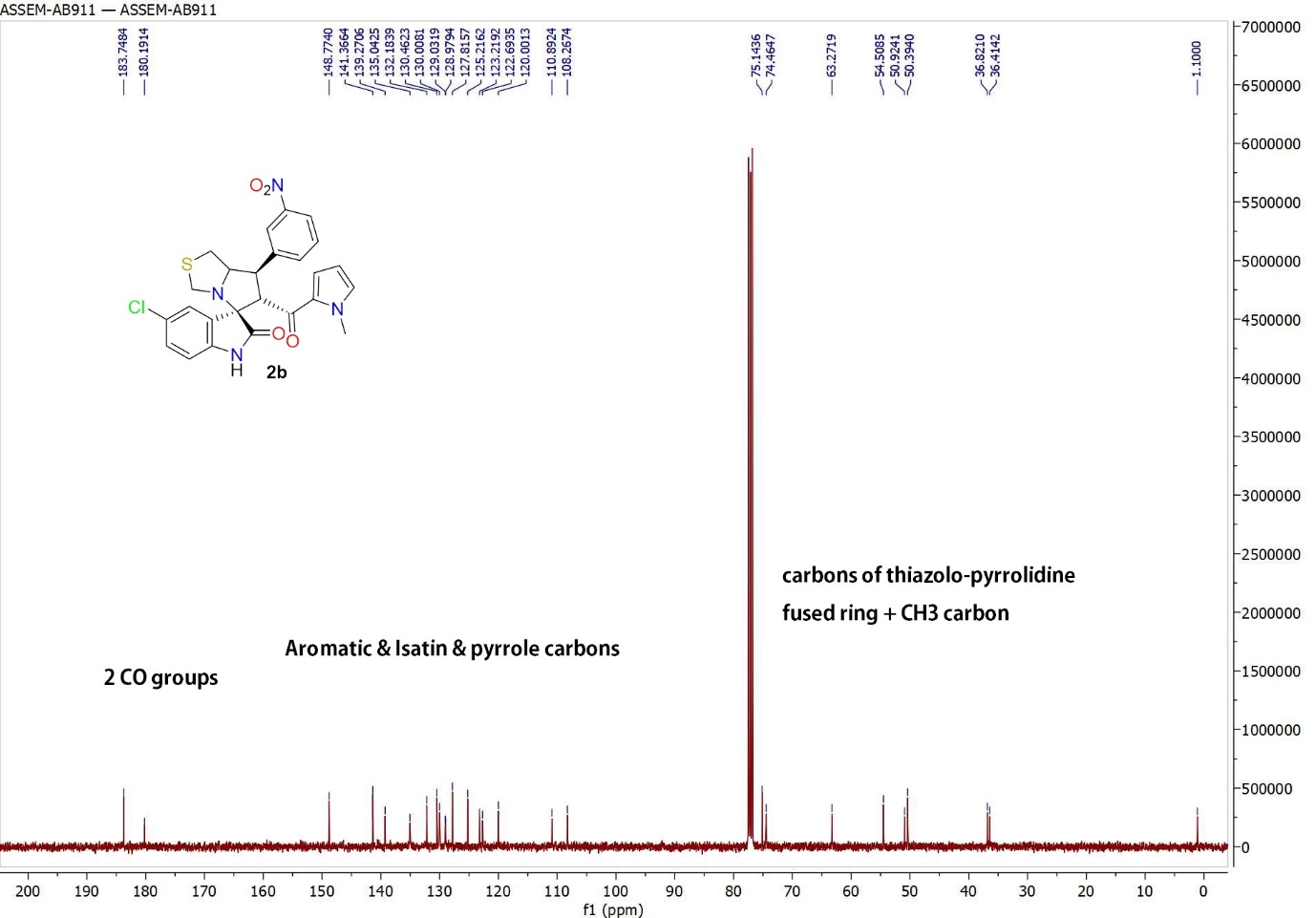


**Figure S6:** ^13^CNMR of **2b**

**
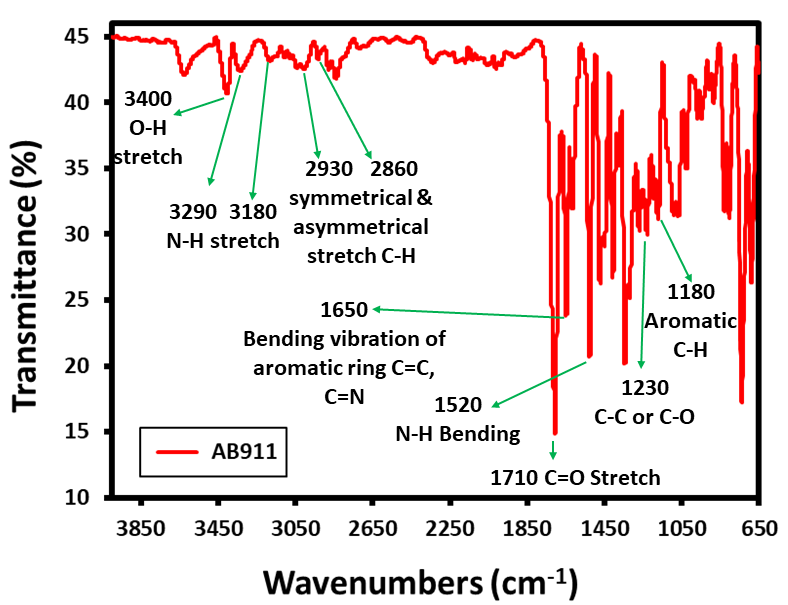
**

**Figure S7:** IR of **2b**


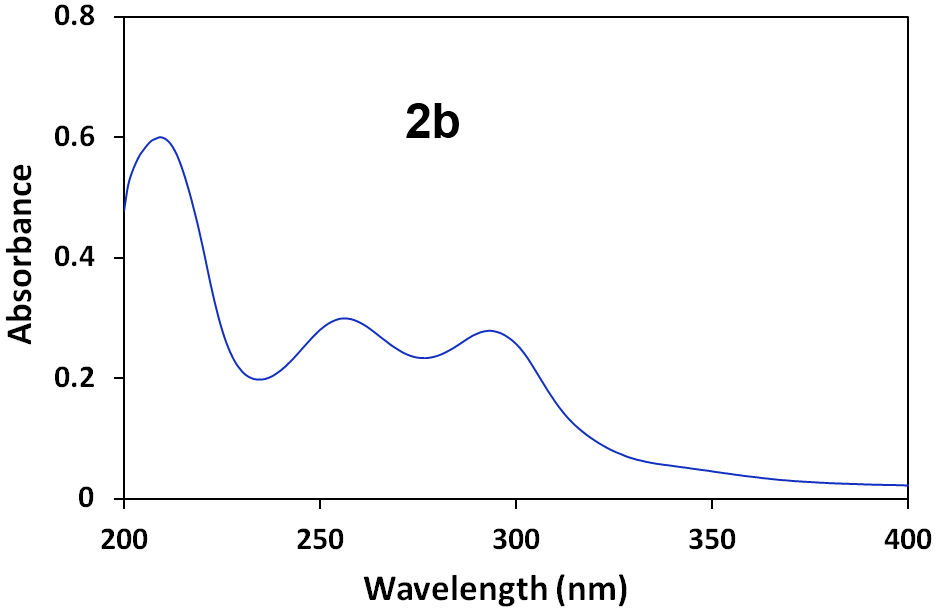


**Figure S8:** UV-Vis of **2b (**in EtOH).

**
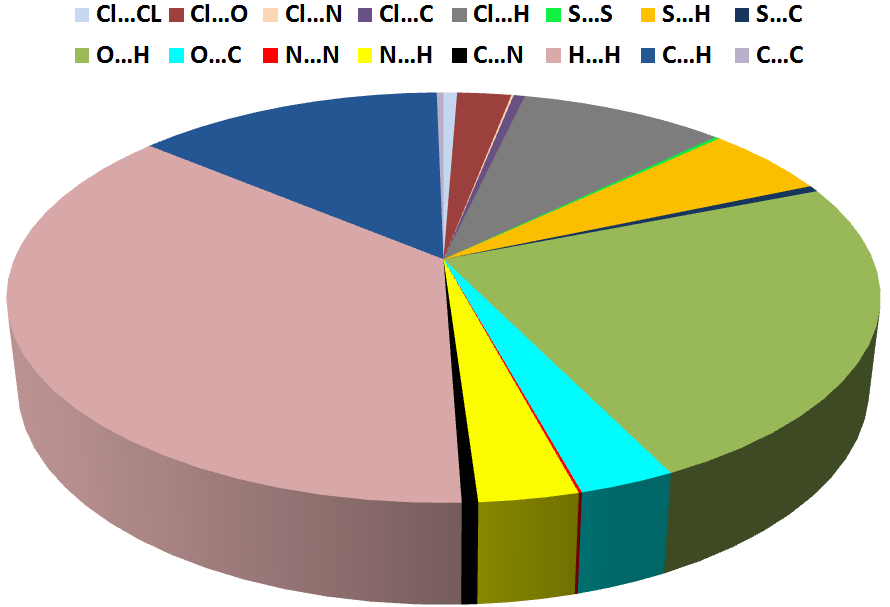
**

**Figure S9:** Summary of the intermolecular interactions and their percentages in the crystal structure of **2b**.


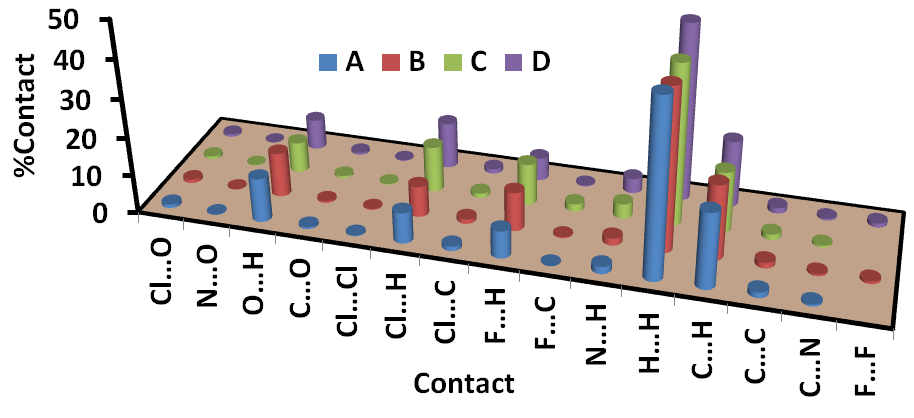


**Figure S10:** The different contacts in **2a.**

| **2a_F1** | **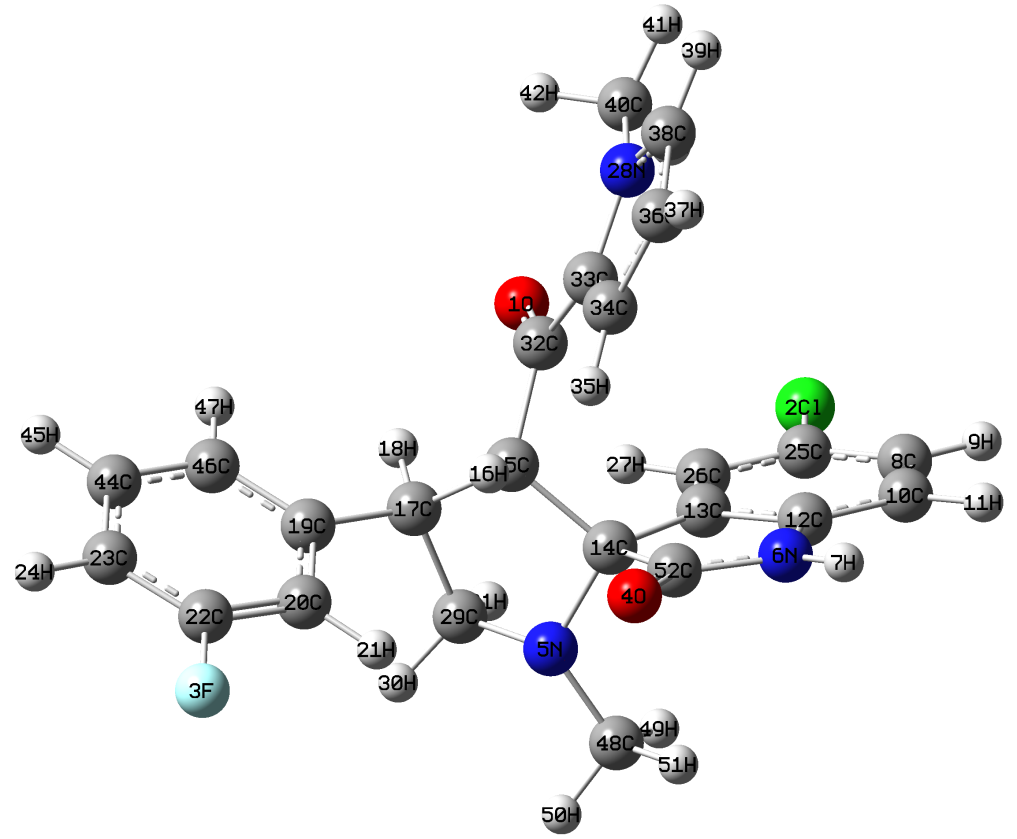** |
| --- | --- |
| **2a_F2** | **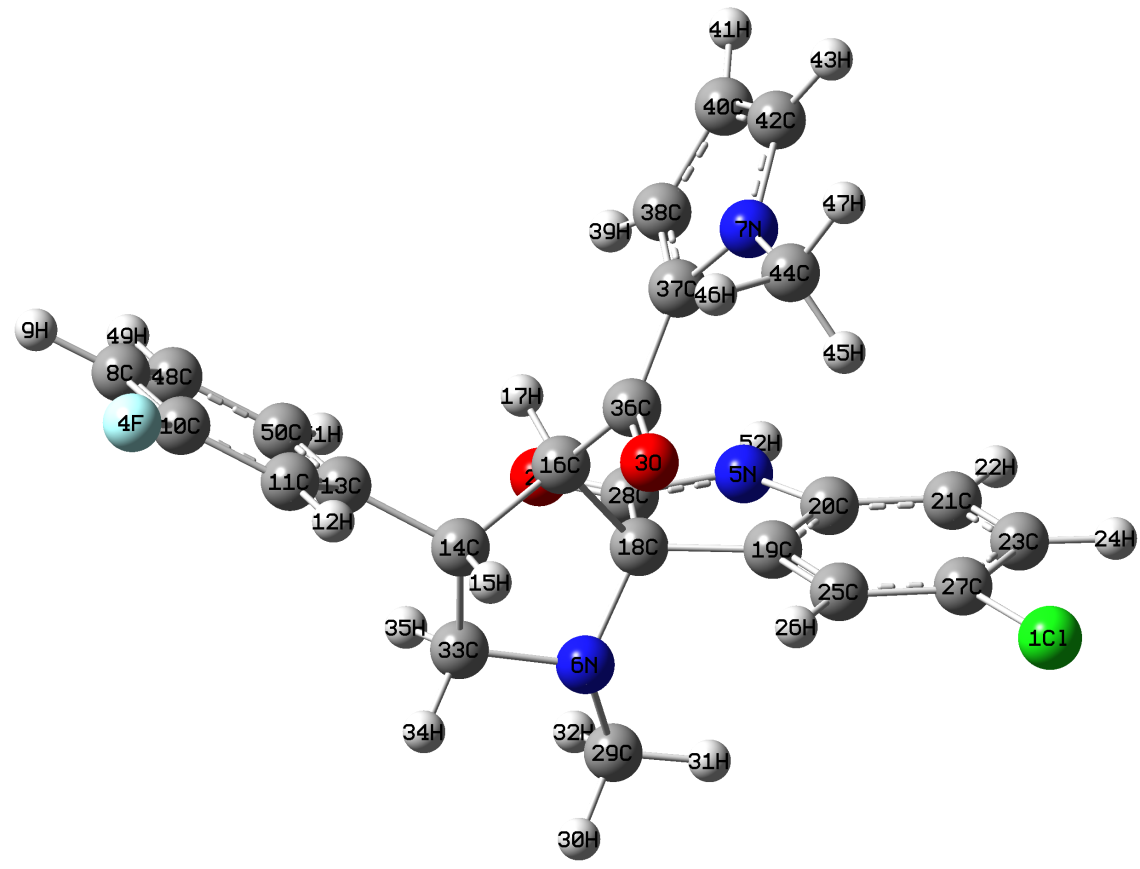** |

**Figure S11.** Optimized molecular geometries with atom numbering for the conformers of **2a**.

| **2b_MeOH** | **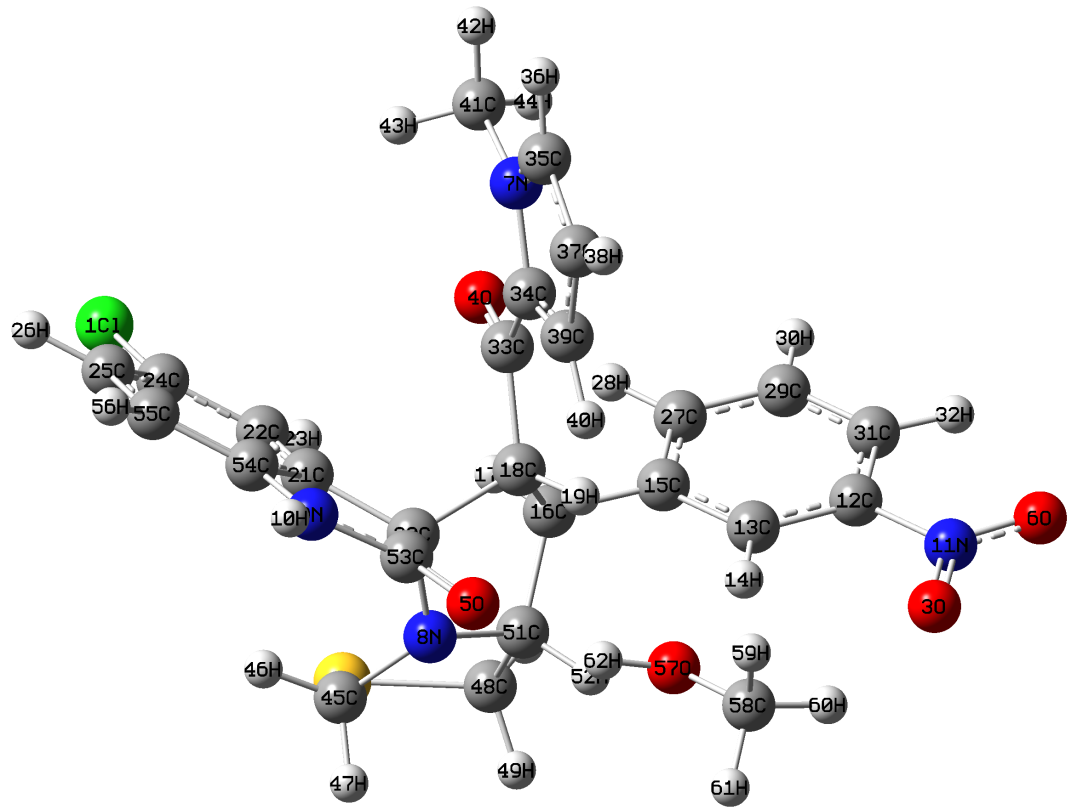** |
| --- | --- |
| **2b_noMeOH** | **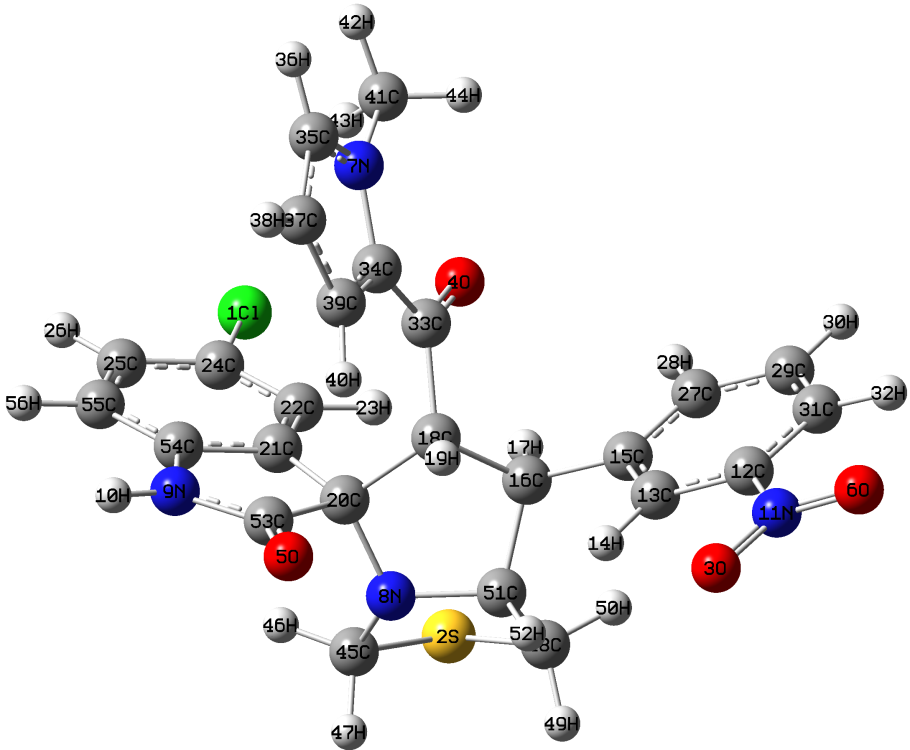** |

**Figure S12.** Optimized molecular geometries with atom numbering for the **2b** with and without the crystallized methanol molecule.

| 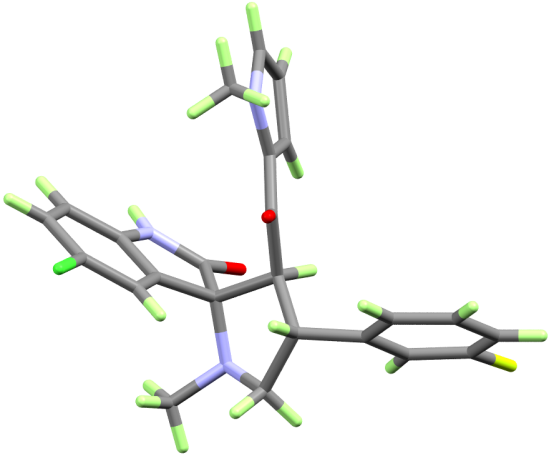 | 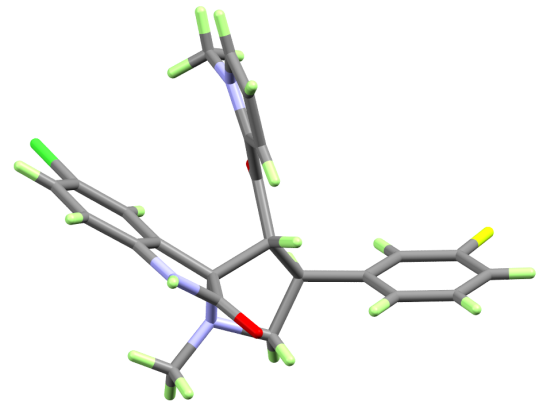 |
| --- | --- |
| 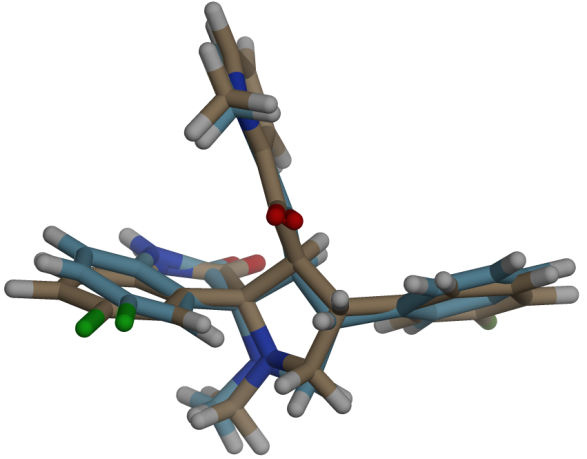 | 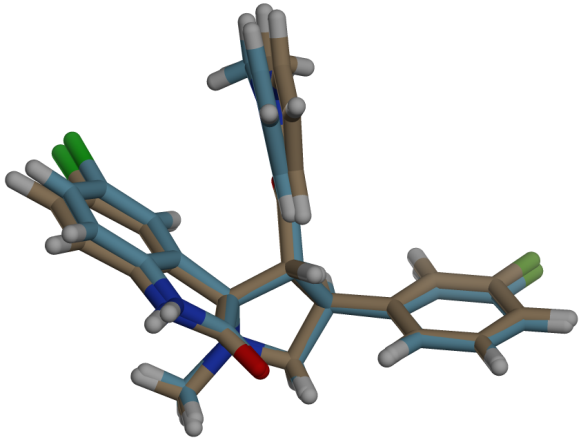 |
| **2a_F1** | **2a_F2** |

**Figure S13:** Structures of conformers **2a_F1** and **2a_F2** and structure matching between the calculated and experimental structures.

| 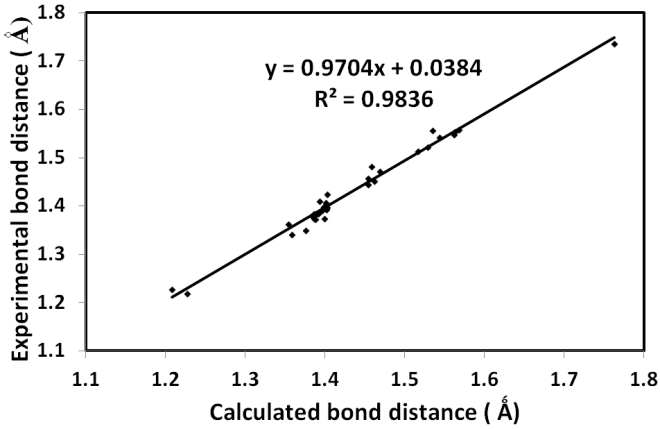 | 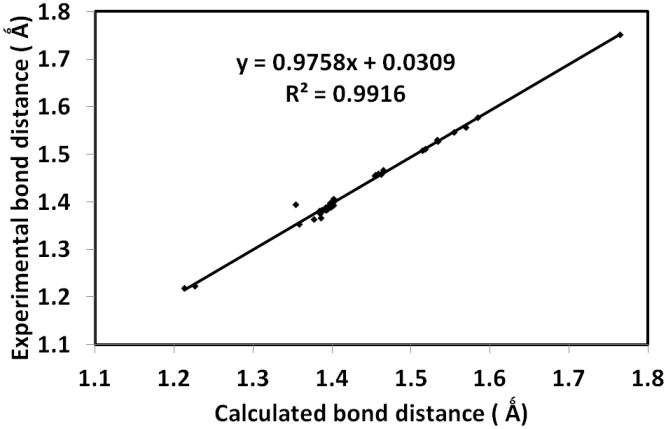 |
| --- | --- |
| **2a_F1** | **2a_F2** |

**Figure S14:** Correlation between the calculated and experimental bond distances of both conformers for **2a**.

| 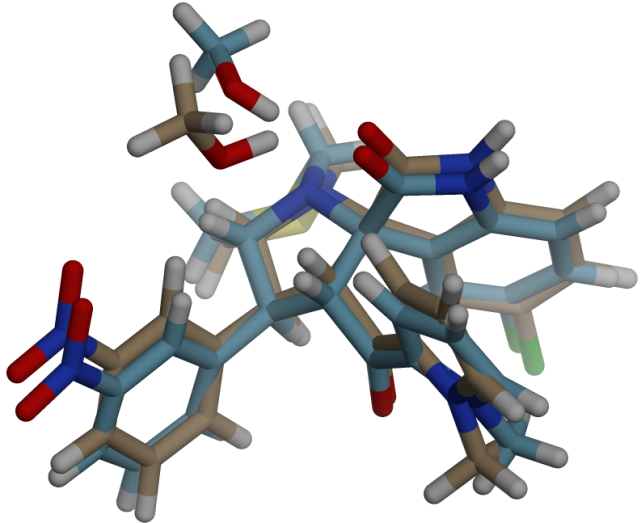 | 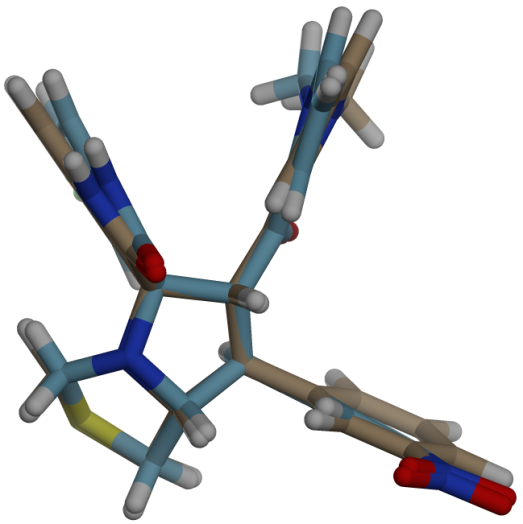 |
| --- | --- |
| 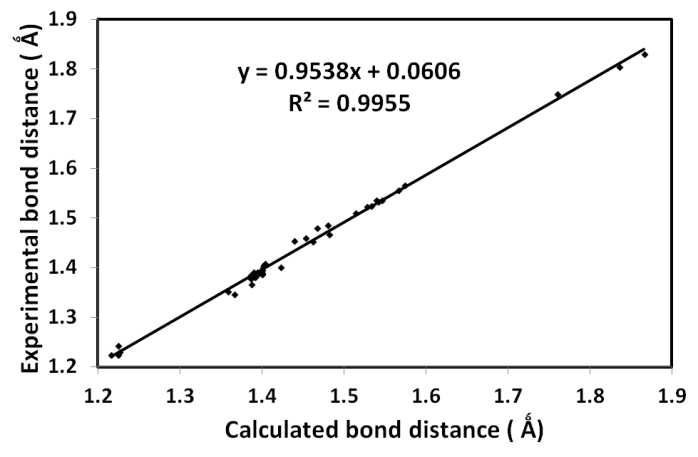 | 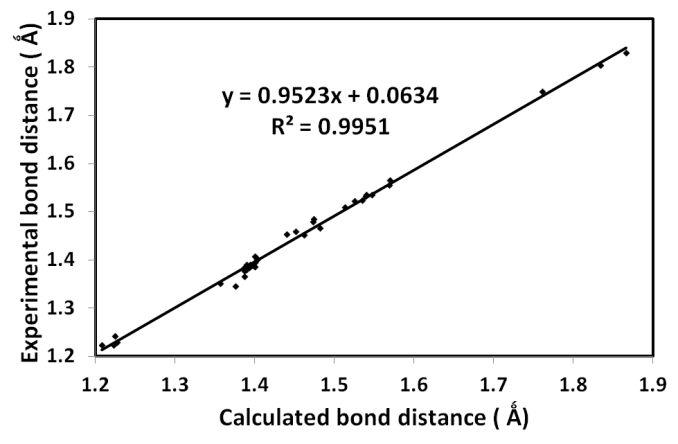 |
| **2b_MeOH** | **2b_no MeOH** |

**Figure S15:** Structures matches between the calculated and experimental structures of **2b** with and without the crystallized methanol molecule and the corresponding correlation graphs between the calculated and experimental bond distances.
